# Grey Wolf Optimization algorithm based on Cauchy-Gaussian mutation and improved search strategy

**DOI:** 10.1038/s41598-022-23713-9

**Published:** 2022-11-08

**Authors:** Kewen Li, Shaohui Li, Zongchao Huang, Min Zhang, Zhifeng Xu

**Affiliations:** College of Computer Science and Technology, China University of Petroleum (East China), Qingdao, 266580 China

**Keywords:** Applied mathematics, Computational science, Computer science, Statistics

## Abstract

The traditional Grey Wolf Optimization algorithm (GWO) has received widespread attention due to features of strong convergence performance, few parameters, and easy implementation. However, in actual optimization projects, there are problems of slow convergence speed and easy to fall into local optimal solution. The paper proposed a Grey Wolf Optimization algorithm based on Cauchy-Gaussian mutation and improved search strategy (CG-GWO) in response to the above problems. The Cauchy-Gaussian mutation operator is introduced to increase the population diversity of the leader wolves and improve the global search ability of the algorithm. This work retains outstanding grey wolf individuals through the greedy selection mechanism to ensure the convergence speed of the algorithm. An improved search strategy was proposed to expand the optimization space of the algorithm and improve the convergence accuracy. Experiments are performed with 16 benchmark functions covering unimodal functions, multimodal functions, and fixed-dimension multimodal functions to verify the effectiveness of the algorithm. Experimental results show that compared with four classic optimization algorithms, particle swarm optimization algorithm (PSO), whale optimization algorithm (WOA), sparrow optimization algorithm (SSA), and farmland fertility algorithm (FFA), the CG-GWO algorithm shows better convergence accuracy, convergence speed, and global search ability. The proposed algorithm shows the same better performance compared with a series of improved algorithms such as the improved grey wolf algorithm (IGWO), modified Grey Wolf Optimization algorithm (mGWO), and the Grey Wolf Optimization algorithm inspired by enhanced leadership (GLF-GWO).

## Introduction

In recent years, swarm intelligence optimization algorithms have been widely applied to optimization problems in various fields due to their flexibility, high robustness, and simple implementation. They are mainly implemented by simulating the predation, migration and other behaviors of various creatures in nature, including Grey Wolf Optimization algorithm^1^ (GWO), particle swarm optimization algorithm^2^ (PSO), whale optimization algorithm^3^ (WOA), and sparrow optimization algorithm^4^ (SSA), etc. Optimization algorithms can effectively improve system efficiency, reduce energy consumption, and help optimizers to use resources rationally. At the same time, this effect becomes more obvious as the scale of optimization problems increase.

The grey wolf optimizer (GWO) is a swarm intelligence optimization algorithm proposed by Mirjalili et al. in 2014, which simulates the group behavior of grey wolves preying on prey and the leadership mechanism. The algorithm is widely used in parameter optimization^5–7^, knapsack problem^8,9^, economic scheduling problem^10–12^, shop scheduling problem^13,14^, fault diagnosis^15–17^, feature selection^18–20^, image processing^21–23^ and many other fields due to its features of few parameters and easy implementation. However, in actual optimization projects, the GWO algorithm has problems of slow convergence speed, insufficient global search ability, and easy to fall into local optimal solution, which has attracted the attention of many scholars and launched a series of studies on Grey Wolf Optimization algorithm. Wen Long et al.^24^ proposed an improved Grey Wolf Optimization algorithm IGWO inspired by particle swarm optimization. The algorithm adds a nonlinear adjustment strategy of the control parameters and a modified position-updating equation based on the personal historical best position and the global best position. Experimental results showed that the algorithm could find more accurate solutions, had a higher convergence speed and fewer fitness function evaluation times. Mittal et al.^25^ paid attention to the proper balance between local search and global search of the GWO algorithm. They proposed the mGWO algorithm by changing the parameter adjustment strategy. Based on the benchmark problems and the WSN clustering problem, it was verified that the algorithm converged fast and had fewer opportunities to get stuck at local minima. The algorithm was very effective for practical applications. Gupta et al.^26^ proposed a Grey Wolf Optimization algorithm GLF-GWO inspired by enhanced leadership. They introduced Levy-flight search mechanism to update the leaders and enhanced the local search ability of the algorithm through the greedy selection mechanism. Experimental results showed that GLF-GWO algorithm had better global search ability to avoid falling into local optimum. Bansal et al.^27^ introduced inverse learning (OBL) to improve the exploration ability of traditional Grey Wolf Optimization algorithm. The proposed algorithm effectively deals with optimization stagnation problems and maintains faster convergence speed. The effectiveness of the algorithm had been proved by experiments. Mirjalili et al.^28^ integrated the archive mechanism and the leader selection mechanism based on the traditional Grey Wolf Optimization algorithm in 2016 and proposed the multi-objective Grey Wolf Optimization algorithm (MOGWO). They experimented on 10 multi-objective benchmark problems to compare with the decomposition-based multi-objective evolutionary algorithm and multi-objective particle swarm algorithm. Experimental results showed that the proposed algorithm was more competitive. Gharehchopogh^29^ used Gaussian mutation, Cauchy mutation, and Levy fight to increase the global search capability of TSA algorithm. Also, QLGCTSA combines the quantum rotation gate to enhance local search capabilities and increase population diversity. Experimental results showed that the QLGCTSA algorithm had outperformed other competing optimization algorithms. The QLGCTSA algorithm is very helpful for our research work. We also used the Cauchy mutation and Gaussian mutation. The differences are as follows: QLGCTSA applied mutation operators to all search agents, while CG-GWO applied the Cauchy-Gaussian mutation to leader wolves. QLGCTSA used mutation operators to improve the global search ability, while CG-GWO used mutation operator to enhance the local search ability and avoid falling into the local optimum. To better clarify the research gaps, we compared the algorithms mentioned above as shown in Table 1.

**Table 1:** A summary of popular algorithms.

Algorithm	References	Improvements
Improved Grey Wolf Optimization algorithm (IGWO)	Wen Long et al.^24^	Nonlinear adjustment strategy of the control parameters
		Modified position-updating equation based on the personal historical best position and the global best position
Modified Grey Wolf Optimization algorithm (mGWO)	Mittal et al.^25^	Pay attention to the proper balance between local search and global search
		Change the parameter adjustment strategy
Enhanced leadership-inspired Grey Wolf Optimization algorithm (GLF-GWO)	Gupta et al.^26^	Levy-flight search mechanism
		Greedy selection mechanism
Opposition-based learning Grey Wolf Optimization algorithm (OBL-GWO)	Bansal et al.^27^	Opposition-based learning
Multi-Objective grey wolf optimizer (MOGWO)	Mirjalili et al.^28^	Archive mechanism
		Leader selection mechanism
An improved tunicate swarm algorithm with best-random mutation strategy (QLGCTSA)	Gharehchopogh^29^	Gaussian mutation
		Cauchy mutation
		Levy-flight search mechanism
		Quantum rotation gate

Because there are problems of slow convergence speed and easy to fall into local optimal solution in the Grey Wolf Optimization algorithm, Grey Wolf Optimization algorithm based on Cauchy-Gaussian mutation and improved search strategy is proposed. The main contributions are as follows:

- We design the Cauchy-Gaussian mutation operator, which acts on the leader wolves. The search range can be increased when the leader wolves tend to the local optimal solution. The operator can effectively improve the local development ability of the leader wolves and avoid falling into the local optimum.
- We propose a greedy selection mechanism^30^, whose main function is to avoid the high diversity of the population caused by variation. The greedy selection mechanism can maintain the diversity of the population and ensure the convergence speed of the algorithm.
- We design an improved search strategy to apply to all grey wolf individuals. This strategy considers the average position of all individuals, which can effectively expand the search space and improve the global search ability of the algorithm.

The rest of this paper is organized as follows: section “Classical Grey Wolf optimizer” provides a brief overview of classical GWO. In section “Proposed method”, the proposed improved algorithm called CG–GWO is discussed in detail. Section “Experimental simulation and result analysis” presents the numerical experimentation and discussion on convergence accuracy, convergence speed, algorithm performance, algorithm runtime and case study of real-world application. The conclusion and future works are presented in section “Conclusion and future works”.

## Classical Grey Wolf optimizer

### Grey Wolf social hierarchy

**Figure 1:**
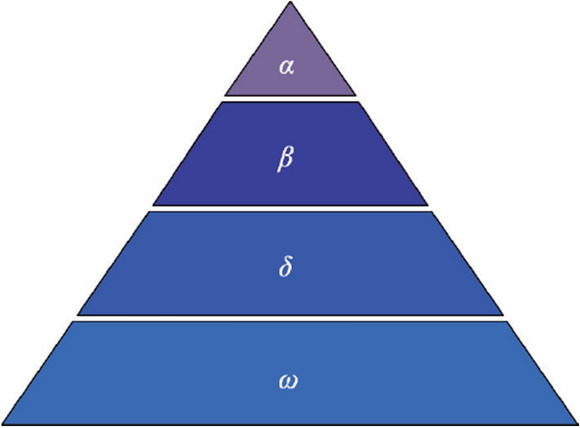
Grey Wolf social hierarchy.

The social hierarchy in the grey wolf population is divided into four levels as shown in Fig. 1. The first level is called *α* wolf, which plays the role of decision maker in the wolves. The wolf has management ability and corresponds to the optimal solution in GWO. The second and third levels are called *β* wolf and *δ* wolf respectively, corresponding to the sub-optimal and third-optimal solutions in GWO. They are mainly responsible for assisting *α* wolf in decision-making, jointly leading and assisting other wolves to keep approaching their prey. The fourth level is called *ω* wolf, which represents other solutions in the optimization process, and updates the position by following the decisions of *α*, *β* and *δ* wolves^31^. During the algorithm iteration, grey wolf individuals of all levels are in a state of competition. After each iteration, the leader wolves must be reselected according to the distance between each grey wolf and the prey.

### Position update

The position update of *α*, *β* and *δ* wolves in the grey wolf population depends on the position of the prey, as shown in formula (1).

1 $$X(t + 1) = X_{p} (t) - A \cdot D$$

where $X(t + 1)$ represents the position of grey wolf after the update, $X_{p} (t)$ represents the position of the prey, *A* is the coefficient vector, and *D* represents the distance between a grey wolf and the prey.

2 $$D = |C \cdot X_{p} (t) - X(t)|$$

3 $$C = 2 \cdot r_{1}$$

4 $$A = 2a \cdot r_{2} - a$$

where $X(t)$ represents the current position of grey wolf, *C* is the coefficient vector, *α* decreases linearly from 2 to 0 over the course of iterations, and $r_{1} ,r_{2}$ are random vectors in [0,1].

According to the social hierarchy of grey wolves, *ω* wolves depend on the leader wolves to update the position. The distance between *ω* wolves and each leader wolves is calculated by formula (5), and finally the direction of movement of the grey wolf individual is determined according to formulas (6) and (7).

5 $$\left\{ \begin{gathered} D_{\alpha } = |C_{1} \cdot X_{\alpha } - X| \hfill \\ D_{\beta } = |C_{2} \cdot X_{\beta } - X| \hfill \\ D_{\delta } = |C_{3} \cdot X_{\delta } - X| \hfill \\ \end{gathered} \right.$$

6 $$\left\{ \begin{gathered} X_{1} = X_{\alpha } - A_{1} \cdot D_{\alpha } \hfill \\ X_{2} = X_{\beta } - A_{2} \cdot D_{\beta } \hfill \\ X_{3} = X_{\delta } - A_{3} \cdot D_{\delta } \hfill \\ \end{gathered} \right.$$

7 $$X(t + 1) = \frac{{X_{1} + X_{2} + X_{3} }}{3}$$

where $X_{\alpha } ,X_{\beta } ,X_{\delta }$ represent positions of *α*, *β* and *δ* wolves respectively, *X* is the current position of the grey wolf individual $D_{\alpha } ,D_{\beta } ,D_{\delta }$ represent the distance between the grey wolf individual and each leader wolves respectively, and $X(t + 1)$ is the position of grey wolf after updating.

### Algorithm flow

**Figure 2:**
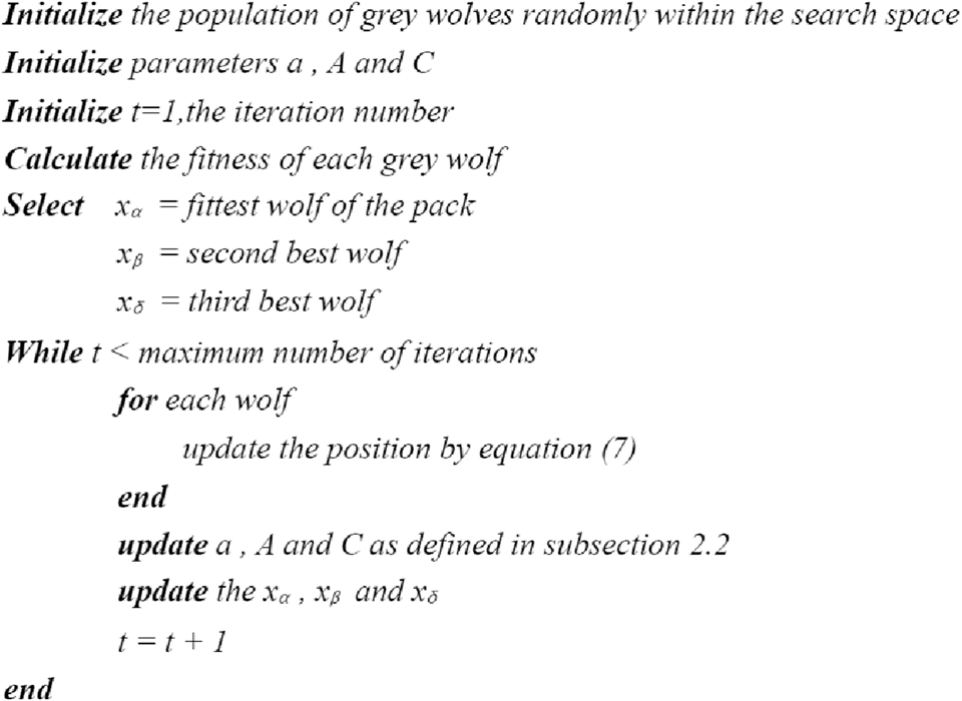
The pseudo code of the traditional GWO algorithm.

The pseudo code of the traditional GWO algorithm is shown in Fig. 2.

## Proposed method

### Cauchy-Gaussian mutation

In the later iterations of the traditional GWO algorithm, grey wolves gradually move closer to *α* wolf, which results in a lack of diversity in the local search of the population and the algorithm tends to converge prematurely. This work introduces the Cauchy-Gaussian mutation operator to improve the diversity of leader wolves and enhance the local search ability in order to solve above problem. After each iteration, *α*, *β* and *δ* wolves are selected for mutation. The mutation and original position are compared based on the greedy selection mechanism. Individuals with better fitness are selected to enter the next iteration. The mathematical definition of the Cauchy-Gaussian mutation strategy is described as follows:

8 $$U_{leader} (t + 1) = X_{leader} \left[ {1 + \lambda_{1} cauchy\left( {0,\sigma^{2} } \right) + \lambda_{2} Gauss\left( {0,\sigma^{2} } \right)} \right]$$

9 $$\sigma = \exp \left( {\frac{{f(X_{leader} ) - f(X_{\alpha } )}}{{|f(X_{\alpha } )|}}} \right)$$

10 $$X(t + 1) = \left\{ \begin{gathered} U_{leader} (t + 1) f(U_{leader} (t + 1)) \le f(X_{leader} ) \hfill \\ X_{leader} otherwise \hfill \\ \end{gathered} \right.$$

where $U_{leader} (t + 1)$ represents the post-mutation position of leader wolves, $X_{leader}$ represents the current position of leader wolves, $cauchy(0,\sigma^{2} )$ is random variables satisfying the Cauchy distribution,$Gauss(0,\sigma^{2} )$ is random variables satisfying the Gaussian distribution, $f(X_{leader} )$ represents the fitness value of leader wolves, $f(X_{\alpha } )$ represents the fitness value of *α* wolf, and $\lambda_{1} ,\lambda_{2}$ are dynamic parameters adaptively adjusted with the number of iterations.

11 $$\lambda_{1} = 1 - \frac{{t^{2} }}{{T^{2} }}$$

12 $$\lambda_{2} = \frac{{t^{2} }}{{T^{2} }}$$

where *t* represents the current iteration number, and *T* represents the maximum number of iterations.

### Improved search strategy

The traditional GWO algorithm has a small number of parameters in the search process and is easy to implement. But the global search ability of the algorithm is weak, resulting in falling into a local optimum in some cases easily. This work proposes an improved search strategy to improve the global search ability of the algorithm and expand the search space. The global search space is expanded under the condition that the current grey wolf individual position $X(t)$ is generated based on the traditional GWO position update formula (7). The mathematical definition is described as follows:

13 $$U(t + 1) = \left\{ \begin{gathered} X_{rand} (t) - r_{1} \times |X_{rand} (t) - 2 \times r_{2} \times X(t)|,r_{5} \ge 0.5 \hfill \\ X_{\alpha } (t) - X_{avg} (t) - r_{3} \times (lb + r_{4} \times (ub - lb)),r_{5} < 0.5 \hfill \\ \end{gathered} \right.$$

where $U(t + 1)$ represents the position of grey wolf individual after through improved search strategy,$X_{rand} (t)$ represents the position of grey wolf individual that is randomly selected from the population at the *t*th iteration,$X(t)$ represents the current position of grey wolf individual,$r_{1} ,r_{2} ,r_{3} ,r_{4} ,r_{5}$ are random vectors in [0,1],$X_{\alpha } (t)$ represents the current position of α wolf,$X_{avg} (t)$ represents the average position of the grey wolf population in the current iteration, and $ub,lb$ are upper and lower bounds of decision variables.

14 $$X(t + 1) = \left\{ \begin{gathered} U(t + 1),f(U(t + 1) \le f(X(t))) \hfill \\ X(t),otherwise \hfill \\ \end{gathered} \right.$$

where $X(t + 1)$ represents the position of grey wolf individual in the $(t + 1)$ th iteration,$f(U(t + 1))$ is the fitness value after updating the position through the improved search strategy, and $f(X(t))$ is the fitness value of the current position.

In the improved search strategy, new solutions generated by iteration are generated around random solutions or optimal solutions, which helps to enhance the search and communication between grey wolf individuals. If the exploration formula (13) cannot provide a better position, the traditional GWO method is used to update the position of the grey wolf individual.

### CG-GWO algorithm

**Figure 3:**
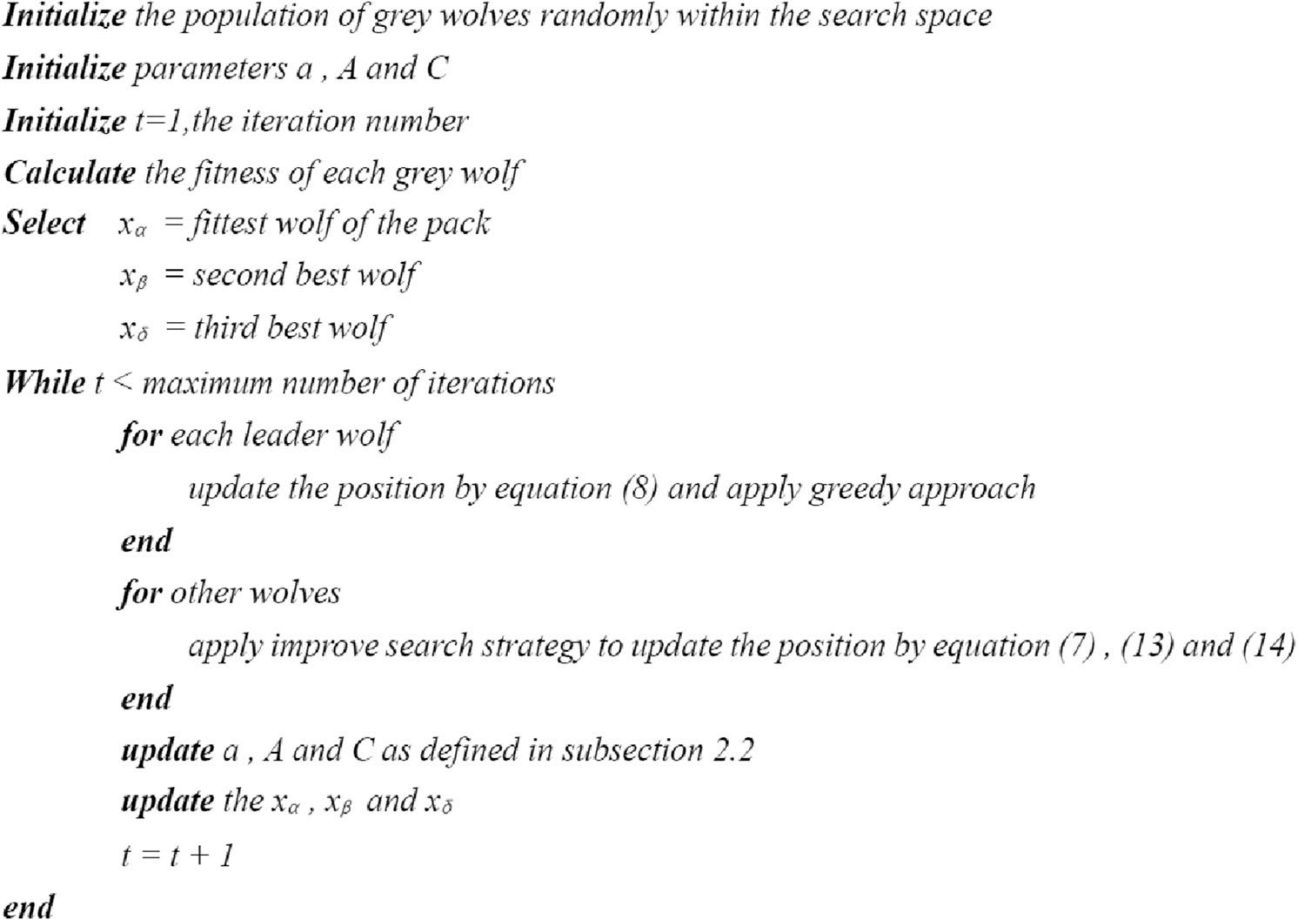
The pseudo code of CG-GWO algorithm.

**Figure 4:**
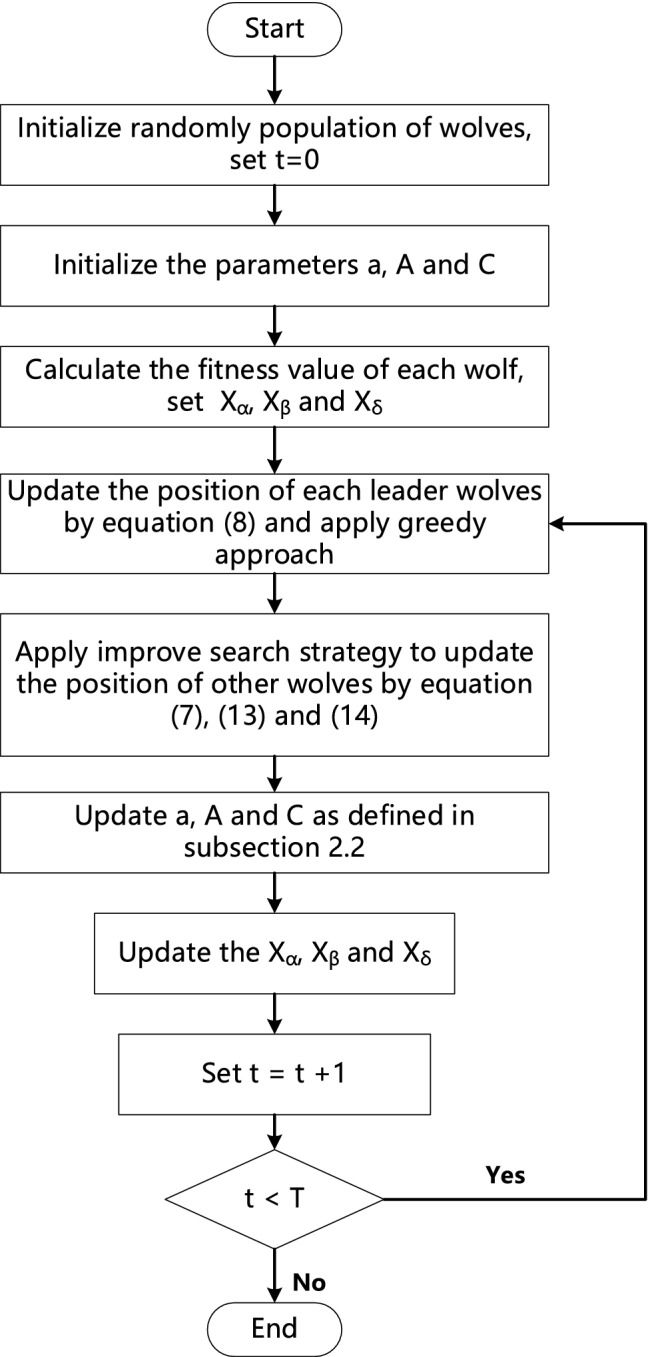
The flow chart of CG-GWO algorithm.

The pseudo code of CG-GWO algorithm is shown in Fig. 3 and the flow chart is shown in Fig. 4.

## Experimental simulation and result analysis

### Experimental design

To verify the performance of our approach, experiments select 16 benchmark functions for simulation experiments, including five unimodal functions (F1–F5) shown in Table 2, six multimodal functions (F6–F11) shown in Table 3, and five fixed-dimension multimodal functions (F12–F16) shown in Table 4. Unimodal functions have only one global optimal solution and no local optimal solution, so they are used to test convergence and exploration abilities of the algorithm. Multimodal functions have only one global optimal solution and others are local optimal solutions. Global search and local optimization abilities of the algorithm are tested through multimodal functions. Fixed-dimension multimodal functions are more complex, which combine multiple basic functions to test the stability of the algorithm.

**Table 2:** Unimodal functions.

Function formula	Dim	Range	f_min_
$F_{1} (x) = \sum\limits_{i = 1}^{n} {x_{i}^{2} }$	30	[− 100, 100]	0
$F_{2} (x) = \sum\limits_{i = 1}^{n} {\left( {\sum\limits_{j = 1}^{i} {x_{j} } } \right)^{2} }$	30	[− 100, 100]	0
$F_{3} (x) = \max \{ |x_{i} |,1 \le i \le n\}$	30	[− 100, 100]	0
$F_{4} (x) = \sum\limits_{i = 1}^{n} {([x_{i} + 0.5])^{2} }$	30	[− 100, 100]	0
$F_{5} (x) = \sum\limits_{i = 1}^{n} {ix_{i}^{4} + random[0,1)}$	30	[− 1.28, 1.28]	0

**Table 3:** Multimodal functions.

Function formula	Dim	Range	f_min_
$F_{6} (x) = \sum\limits_{i = 1}^{n} { - x_{i} \sin \left( {\sqrt {|x_{i} |} } \right)}$	30	[− 500, 500]	− 418.9829 × 5
$F_{7} (x) = \sum\limits_{i = 1}^{n} {\left[ {x_{i}^{2} - 10\cos (2\pi x_{i} ) + 10} \right]}$	30	[− 5.12, 5.12]	0
$$\begin{aligned} F_{8} (x) & = - 20\exp \left( { - 0.2\sqrt {\frac{1}{n}\sum\limits_{i = 1}^{n} {x_{i}^{2} } } } \right) \\ & \;\;\; - \exp \left( {\frac{1}{n}\sum\limits_{i = 1}^{n} {\cos (2\pi x_{i} )} } \right) + 20 + e \\ \end{aligned}$$	30	[− 32, 32]	0
$F_{9} (x) = \frac{1}{4000}\sum\limits_{i = 1}^{n} {x_{i}^{2} } - \prod\limits_{i = 1}^{n} {\cos \left( {\frac{{x_{i} }}{\sqrt i }} \right)} + 1$	30	[− 30, 30]	0
$$\begin{aligned} F_{10} (x) & = 0.1\left\{ {\sin^{2} (3\pi x_{1} )} \right. + \sum\limits_{i = 1}^{n} {(x_{i} - 1)^{2} \left[ {1 + \sin^{2} (3\pi x_{i} + 1)} \right]} \\ & \;\;\; + \left. {(x_{n} - 1)^{2} \left( {1 + \sin^{2} (2\pi x_{n} )} \right)} \right\} + \sum\limits_{i = 1}^{n} {u(x_{i} ,5,100,4)} \\ \end{aligned}$$	30	[− 50, 50]	0
$$\begin{aligned} F_{11} (x) & = \frac{\pi }{n}\left\{ {10\sin (\pi y_{1} ) + \sum\limits_{i = 1}^{n - 1} {(y_{i} - 1)^{2} \left[ {1 + 10\sin^{2} (\pi y_{i + 1} )} \right]} } \right. \\ & \;\;\;\left. { + (y_{n} - 1)^{2} } \right\} + \sum\limits_{i = 1}^{n} {u(x_{i} ,10,100,4)} \\ y_{i} & = 1 + \frac{{x_{i} + 1}}{4}u(x_{i} ,a,k,m) = \left\{ \begin{gathered} k(x_{i} - a)^{m} x_{i} > a \hfill \\ 0 - a < x_{i} < a \hfill \\ k( - x_{i} - a)^{m} x_{i} < - a \hfill \\ \end{gathered} \right. \\ \end{aligned}$$	30	[− 50, 50]	0

**Table 4:** Fixed-dimension multimodal functions.

Function formula	Dim	Range	f_min_
$F_{12} (x) = \left( {\frac{1}{500} + \sum\limits_{j = 1}^{25} {\frac{1}{{j + \sum\limits_{i = 1}^{2} {(x_{i} - a_{ij} )^{6} } }}} } \right)^{ - 1}$	2	[− 65, 65]	1
$F_{13} (x) = \sum\limits_{i = 1}^{11} {\left[ {a_{i} - \frac{{x_{1} \left( {b_{i}^{2} + b_{i} x_{2} } \right)}}{{b_{i}^{2} + b_{i} x_{3} + x_{4} }}} \right]^{2} }$	4	[− 5, 5]	0.0003
$F_{14} (x) = 4x_{1}^{2} - 2.1x_{1}^{4} + \frac{1}{3}x_{1}^{6} + x_{1} x_{2} - 4x_{2}^{2} + 4x_{2}^{4}$	2	[− 5, 5]	− 1.0316
$F_{15} (x) = \left( {x_{2} - \frac{5.1}{{4\pi^{2} }}x_{1}^{2} + \frac{5}{\pi }x_{1} - 6} \right)^{2} + 10\left( {1 - \frac{1}{8\pi }} \right)\cos x_{1} + 10$	2	[− 5, 5]	0.398
$$\begin{aligned} F_{16} (x) & = \left[ {1 + (x_{1} + x_{2} + 1)^{2} \left( {19 - 14x_{1} + 3x_{1}^{2} - 14x_{2} + 6x_{1} x_{2} + 3x_{2}^{2} } \right)} \right] \\ & \;\;\; \times \left[ {30 + \left( {2x_{1} - 3x_{2} } \right)^{2} \times \left( {18 - 32x_{1} + 12x_{1}^{2} + 48x_{2} - 36x_{1} x_{2} + 27x_{2}^{2} } \right)} \right] \\ \end{aligned}$$	2	[− 2, 2]	3

This work conducts two-part comparative experiments to test the effectiveness of our approach. On the one hand, the proposed algorithm is compared with classic optimization algorithms such as traditional Grey Wolf Optimization algorithm (GWO), particle swarm optimization algorithm (PSO), whale optimization algorithm (WOA), sparrow optimization algorithm (SSA) and farmland fertility algorithm (FFA)^32^. On the other hand, the proposed algorithm is compared with a series of improved algorithms such as the enhanced leadership inspired Grey Wolf Optimization algorithm (GLF-GWO), inspired Grey Wolf Optimization algorithm (IGWO) and modified Grey Wolf Optimization algorithm (mGWO).

Experiments in this work are all implemented on a PC (8G memory, 903G hard disk, CPU: Intel i7-4790) using python 3.6.8 environment. To ensure the fairness of the experiments, all algorithms are independently run 30 times on each function. The population size is set to 30 and the maximum number of iterations is 200. Finally, the optimal value (Best), average value (Ave), worst value (Worst) and standard value (SD) of all benchmark functions are obtained.

### Experimental results and analysis

#### Convergence accuracy analysis

To verify the optimization effect of our approach on the accuracy of convergence, it is compared with algorithms such as GWO, PSO, WOA, SSA, FFA, IGWO, mGWO and GLF-GWO on 16 benchmark functions in simulation experiments. Experimental results are presented in Tables 5 and 6. The bold part indicates relatively superior comparison results.

**Table 5:** Experimental results comparison of classical optimization algorithms.

Function	Algorithm	Best	Ave	Worst	SD
F1	PSO	2.42E−03	2.03E−02	6.91E−02	1.58E−02
	WOA	6.06E−43	1.29E−38	1.05E−37	2.56E−38
	SSA	1.18E−01	4.12E+00	1.75E+01	4.30E+00
	FFA	3.42E−23	4.62E−14	8.41E−06	1.52E−08
	GWO	7.78E−17	1.35E−15	4.07E−15	1.07E−15
	**CG-GWO**	**1.49E**−**162**	**2.81E**−**150**	**5.43E**−**149**	**1.02E**−**149**
F2	PSO	3.63E+01	1.31E+02	2.60E+02	4.71E+01
	WOA	1.88E+04	4.13E+04	5.99E+04	1.12E+04
	SSA	7.63E+02	1.89E+03	4.14E+03	8.90E+02
	FFA	3.52E+03	4.25E+03	6.51E+03	2.35E+02
	GWO	3.48E−04	3.05E−02	2.83E−01	5.93E−02
	**CG-GWO**	**1.37E**−**142**	**3.56E**−**133**	**8.95E**−**132**	**1.60E**−**132**
F3	PSO	8.94E**−**01	1.32E+00	1.73E+00	2.02E−01
	WOA	6.09E−04	3.20E+01	7.74E+01	2.44E+01
	SSA	5.83E+00	1.13E+01	1.87E+01	3.43E+00
	FFA	4.34E−02	6.15E−01	3.15E−01	2.05E−01
	GWO	1.24E−04	9.01E−04	3.11E−03	7.08E−04
	**CG-GWO**	**1.01E**−**77**	**1.04E**−**72**	**2.26E**−**71**	**4.14E**−**72**
F4	PSO	3.35E−03	1.82E−02	5.67E−02	1.34E−02
	WOA	2.98E−02	1.36E−01	3.49E−01	8.62E−02
	SSA	1.82E−01	3.94E+00	1.79E+01	4.09E+00
	FFA	3.15E−02	5.16E−01	6.59E−02	1.63E−01
	GWO	1.32E−04	2.45E−01	7.58E−01	2.44E−01
	**CG-GWO**	**7.42E**−**05**	**1.51E**−**05**	**2.10E**−**05**	**3.28E**−**05**
F5	PSO	6.68E−02	8.95E−01	2.87E+00	1.15E+00
	WOA	5.91E−05	1.83E−03	8.91E−03	2.20E−03
	SSA	9.15E−02	1.82E−01	4.34E−01	6.61E−02
	FFA	3.48E−02	4.15E−02	5.95E−02	1.68E−01
	GWO	6.27E−04	2.13E−03	5.11E−03	1.08E−03
	**CG-GWO**	**4.76E**−**05**	**2.51E-04**	**6.91E-04**	**1.85E**−**04**
F6	PSO	− 5.38E+03	− 4.40E + 03	− 3.50E+03	5.50E+02
	WOA	**− 1.26E+04**	− 1.09E + 04	− 7.65E+03	1.60E+03
	SSA	− 8.64E+03	− 7.31E+03	− 5.82E+03	6.71E+02
	FFA	− 7.62+03	− 6.82E+03	− 5.16E+03	1.51E+02
	GWO	− 8.07E+03	− 6.30E+03	− 3.04E+03	1.38E+03
	**CG-GWO**	**− 1.26E+04**	− **1.26E+04**	− **1.25E+04**	**4.50E+00**
F7	PSO	5.92E+01	1.07E+02	1.73E + 02	2.73E+01
	WOA	**0**	1.89E−15	5.68E−14	1.02E−14
	SSA	1.82E+01	3.90E+01	7.16E+01	1.32E+01
	FFA	1.23E+01	2.61E+01	6.15E+01	1.22E+01
	GWO	2.25E+00	1.33E+01	4.22E+01	8.17E+00
	**CG-GWO**	**0**	**0**	**0**	**0**
F8	PSO	3.44E−02	3.64E−01	1.55E+00	4.15E−01
	WOA	**4.44E−16**	6.96E−15	2.18E−14	4.41E−15
	SSA	2.05E+00	3.53E+00	5.92E+00	9.28E−01
	FFA	3.51E−01	4.34E−01	5.16E−01	5.15E−01
	GWO	3.28E−09	9.36E−09	2.07E−08	4.34E−09
	**CG-GWO**	**4.44E−16**	**4.44E−16**	**4.44E−16**	**1.48E−31**
F9	PSO	2.33E−02	9.22E−01	2.08E+00	5.38E−01
	WOA	**0**	1.59E−02	3.24E−01	6.34E−02
	SSA	3.38E−01	8.84E−01	1.10E+00	2.26E−01
	FFA	3.18E−01	5.68E−01	6.15E−01	1.25E−01
	GWO	2.66E−15	3.31E−03	3.69E−02	7.80E−03
	**CG-GWO**	**0**	**0**	**0**	**0**
F10	PSO	1.15E−03	1.31E−02	4.44E−02	1.10E−02
	WOA	6.09E−02	1.94E−01	3.61E−01	7.57E−02
	SSA	4.97E+00	5.13E+01	6.36E+01	1.34E+01
	FFA	4.53E−01	6.15E−01	2.54E−01	5.16E−01
	GWO	1.94E−04	2.36E−01	5.30E−01	1.41E−01
	**CG-GWO**	**6.43E−05**	**1.16E−04**	**1.79E−04**	**3.21E−05**
F11	PSO	1.35E−01	3.58E−01	5.15E+00	2.15E−01
	WOA	6.59E−04	7.12E−04	3.15E−03	3.15E−03
	SSA	3.21E−03	5.15E−03	2.15E−02	1.25E−03
	FFA	6.52E−04	7.36E−04	9.15E−04	1.42E−04
	GWO	2.48E−02	3.56E−03	4.16E−02	2.18E−02
	**CGGWO**	**6.25E−08**	**3.36E−07**	**7.56E−07**	**1.59E−08**
F12	PSO	**9.98E−01**	1.36E+00	1.99E+00	4.79E**−**01
	WOA	**9.98E−01**	1.89E+00	1.08E+01	1.83E+00
	SSA	**9.98E−01**	1.59E+00	5.93E+00	1.04E+00
	FFA	**9.98E−01**	1.68E+00	3.51E+00	1.82E+00
	GWO	**9.98E−01**	2.70E+00	1.08E+01	2.84E+00
	**CG-GWO**	**9.98E−01**	**9.98E−01**	**9.98E−01**	**1.79E−10**
F13	PSO	3.31E−04	2.75E−03	2.04E−02	5.88E−03
	WOA	3.13E−04	7.03E−04	1.65E−03	4.02E−04
	SSA	4.88E−04	1.69E−03	2.04E−02	3.49E−03
	FFA	3.84E−04	3.45E−03	1.89E−02	4.16E−03
	GWO	3.08E−04	1.72E−03	2.04E−02	4.99E−03
	**CG-GWO**	**3.07E−04**	**3.74E−04**	**1.22E−03**	**1.98E−04**
F14	PSO	**− 1.03E+00**	**− 1.03E+00**	**− 1.03E+00**	6.66E−16
	WOA	**− 1.03E+00**	**− 1.03E+00**	**− 1.03E+00**	8.28E−10
	SSA	**− 1.03E+00**	**− 1.03E+00**	**− 1.03E+00**	9.92E−14
	FFA	**− 1.03E+00**	**− 1.03E+00**	**− 1.03E+00**	7.84E−11
	GWO	**− 1.03E+00**	**− 1.03E+00**	**− 1.03E+00**	5.53E−08
	CG-GWO	**− 1.03E+00**	**− 1.03E+00**	**− 1.03E+00**	**4.74E**−**18**
F15	PSO	**3.98E**−**01**	**3.98E**−**01**	**3.98E**−**01**	1.11E−15
	WOA	**3.98E**−**01**	**3.98E**−**01**	**3.98E**−**01**	1.15E−06
	SSA	**3.98E**−**01**	**3.98E**−**01**	**3.98E**−**01**	2.95E−14
	FFA	**3.98E**−**01**	**3.98E**−**01**	**3.98E**−**01**	2.62E−05
	GWO	**3.98E**−**01**	**3.98E**−**01**	**3.98E**−**01**	1.78E−06
	CG-GWO	**3.98E**−**01**	**3.98E**−**01**	**3.98E**−**01**	**2.05E**−**16**
F16	PSO	**3.00E+00**	**3.00E+00**	**3.00E+00**	1.92E−15
	WOA	**3.00E+00**	**3.00E+00**	**3.00E+00**	2.11E−05
	SSA	**3.00E+00**	**3.00E+00**	**3.00E+00**	6.02E−13
	FFA	**3.00E+00**	**3.00E+00**	**3.00E+00**	5.71E−10
	GWO	**3.00E+00**	**3.00E+00**	**3.00E+00**	2.38E−05
	CG-GWO	**3.00E+00**	**3.00E+00**	**3.00E+00**	**7.33E**−**16**

**Table 6:** Experimental results comparison of improved GWO algorithms.

Function	Algorithm	Best	Ave	Worst	SD
F1	GWO	7.78E−17	1.35E−15	4.07E−15	1.07E−15
	mGWO	1.03E−21	1.20E−20	4.06E−20	1.14E−20
	IGWO	4.81E−25	9.61E−24	4.29E−23	1.00E−23
	GLF-GWO	3.81E−33	2.85E−31	1.99E−30	4.28E−31
	**CG-GWO**	**1.49E**−**162**	**2.81E**−**150**	**5.43E**−**149**	**1.02E**−**149**
F2	GWO	3.48E−04	3.05E−02	2.83E−01	5.93E−02
	mGWO	1.95E−05	2.96E−03	2.68E−02	6.17E−03
	IGWO	9.61E−07	9.13E−04	1.98E−02	3.55E−03
	GLF-GWO	2.34E−04	7.66E−03	6.57E−02	1.42E−02
	**CG-GWO**	**1.37E**−**142**	**3.56E**−**133**	**8.95E**−**132**	**1.60E**−**132**
F3	GWO	1.24E−04	9.01E−04	3.11E−03	7.08E−04
	mGWO	6.90E−06	3.61E−05	1.02E−04	2.77E−05
	IGWO	5.39E−06	3.73E−05	1.74E−04	3.80E−05
	GLF-GWO	3.95E−05	1.44E−04	3.35E−04	8.01E−05
	**CG-GWO**	**1.01E**−**77**	**1.04E−72**	**2.26E**−**71**	**4.14E**−**72**
F4	GWO	1.32E−04	2.45E−01	7.58E−01	2.44E−01
	mGWO	4.39E−04	2.96E−01	1.00E+00	2.25E−01
	IGWO	3.24E−01	8.71E−01	1.60E+00	3.75E−01
	GLF-GWO	1.10E−04	7.77E−04	3.86E−04	1.85E−04
	**CG-GWO**	**7.42E**−**05**	**1.51E−05**	**2.10E**−**05**	**3.28E**−**05**
F5	GWO	6.27E−04	2.13E−03	5.11E−03	1.08E−03
	mGWO	4.09E−04	1.67E−03	4.60E−03	9.45E−04
	IGWO	7.22E−04	1.74E−03	4.28E−03	8.12E−04
	GLF-GWO	9.02E−04	2.90E−03	6.73E−03	1.42E−03
	**CG-GWO**	**4.76E**−**05**	**2.51E−04**	**6.91E−04**	**1.85E**−**04**
F6	GWO	− 8.07E+03	− 6.30E+03	− 3.04E+03	1.38E+03
	mGWO	− 8.00E+03	− 5.92E+03	− 3.26E+03	1.46E+03
	IGWO	− 4.45E+03	− 3.80E+03	− 3.12E+03	3.55E+02
	GLF-GWO	− 1.00E+04	− 8.81E+03	− 5.49E+03	9.63E +02
	**CG-GWO**	− **1.26E+04**	− **1.26E+04**	− **1.25E+04**	**4.50E +00**
F7	GWO	2.25E+00	1.33E+01	4.22E +01	8.17E +00
	mGWO	4.55E−13	8.52E+00	4.37E +01	1.03E +01
	IGWO	5.41E+00	4.18E+01	9.41E +01	2.23E +01
	GLF-GWO	5.68E−14	2.82E+00	1.51E +01	3.65E +00
	**CG-GWO**	**0**	**0**	**0**	**0**
F8	GWO	3.28E−09	9.36E−09	2.07E−08	4.34E−09
	mGWO	5.64E−12	2.20E−11	7.32E−11	1.64E−11
	IGWO	1.57E−13	6.29E−13	2.55E−12	4.77E−13
	GLF-GWO	3.60E−14	4.51E−14	6.44E−14	5.78E−15
	**CG-GWO**	**4.44E−16**	**4.44E−16**	**4.44E−16**	**1.48E**−**31**
F9	GWO	2.66E−15	3.31E−03	3.69E−02	7.80E−03
	mGWO	**0**	1.98E−03	1.54E−02	5.04E−03
	IGWO	**0**	6.76E−03	1.77E−02	6.94E−03
	GLF-GWO	**0**	4.77E−03	4.92E−02	1.07E−02
	**CG-GWO**	**0**	**0**	**0**	**0**
F10	GWO	1.94E−04	2.36E**−**01	5.30E**−**01	1.41E−01
	mGWO	6.68E−04	2.04E**−**01	4.52E**−**01	1.15E−01
	IGWO	3.31E−01	8.43E**−**01	1.49E +00	2.87E−01
	GLF-GWO	6.57E−05	4.08E**−**04	1.10E**−**02	1.97E−03
	**CG-GWO**	**6.43E−05**	**1.16E−04**	**1.79E−04**	**3.21E**−**05**
F11	GWO	2.48E**−**02	3.56E**−**03	4.16E**−**02	2.18E−02
	mGWO	1.25E**−**06	5.15E**−**06	5.15E**−**05	2.15E−06
	IGWO	3.12E**−**06	6.15E**−**06	3.51E**−**05	6.18E**−**05
	GLF-GWO	6.52E**−**07	7.19E**−**07	1.25E**−**-06	1.54E**−**06
	**CG-GWO**	**6.25E−08**	**3.36E−07**	**7.56E-07**	**1.59E−08**
F12	GWO	**9.98E−01**	2.70E +00	1.08E +01	2.84E +00
	mGWO	**9.98E−01**	2.25E +00	1.08E +01	1.86E +00
	IGWO	**9.98E−-01**	4.00E +00	1.27E +01	3.82E +00
	GLF-GWO	**9.98E−01**	**9.98E−01**	**9.98E−01**	2.11E**−**10
	**CG-GWO**	**9.98E−01**	**9.98E−01**	**9.98E−01**	**1.79E−10**
F13	GWO	3.08E**−**04	1.72E**−**03	2.04E**−**02	4.99E**−**03
	mGWO	3.08E**−**04	3.11E**−**03	2.04E**−**02	6.77E**−**03
	IGWO	3.08E**−**04	1.09E**−**03	2.09E**−**02	3.69E**−**03
	GLF-GWO	**3.07E−04**	1.80E**−**03	2.04E**−**02	4.97E**−**04
	**CG-GWO**	**3.07E−04**	**3.74E−04**	**1.22E−03**	**1.98E−04**
F14	GWO	− **1.03E+00**	− **1.03E +00**	− **1.03E +00**	5.53E−08
	mGWO	− **1.03E+00**	− **1.03E +00**	− **1.03E +00**	2.06E−07
	IGWO	− **1.03E+00**	− **1.03E +00**	− **1.03E +00**	2.10E−06
	GLF-GWO	**− 1.03E+00**	− **1.03E +00**	− **1.03E +00**	2.81E−08
	CG-GWO	**− 1.03E+00**	− **1.03E +00**	− **1.03E+00**	**4.74E**−**18**
F15	GWO	**3.98E**−**01**	**3.98E**−**01**	**3.98E**−**01**	1.78E−06
	mGWO	**3.98E**−**01**	**3.98E**−**01**	**3.98E**−**01**	7.00E−06
	IGWO	**3.98E**−**01**	**3.98E**−**01**	**3.98E**−**01**	1.11E−04
	GLF-GWO	**3.98E**−**01**	**3.98E**−**01**	**3.98E**−**01**	1.20E−06
	CG-GWO	**3.98E**−**01**	**3.98E**−**01**	**3.98E**−**01**	**2.05E**−**16**
F16	GWO	**3.00E+00**	**3.00E+00**	**3.00E+00**	2.38E−05
	mGWO	**3.00E+00**	**3.00E+00**	**3.00E+00**	1.96E−05
	IGWO	**3.00E+00**	**3.00E+00**	**3.00E+00**	1.27E−05
	GLF-GWO	**3.00E+00**	**3.00E+00**	**3.00E+00**	2.14E−06
	CG-GWO	**3.00E+00**	**3.00E+00**	**3.00E+00**	**7.33E**−**16**

As shown in Table 5, statistics of the optimal value, average value and worst value of the CG-GWO algorithm on all benchmark functions were at the optimal level. Experiments were given the same initial population size and number of iterations. On the benchmark function F12, our approach and other four classical optimization algorithms in the experiment could all find the optimal value. However, the average value, worst value and standard deviation of our approach were significantly better. In contrast, on the benchmark functions F1, F2, F3, F4, F7, F9, and F10, our approach had shown absolute superiority compared with other classic optimization algorithms in the experiment. All statistics were several orders of magnitude higher. On the benchmark functions F5 and F13, although the effect of our approach was not so obvious, it was better than other four classic optimization algorithms in the experiment in terms of all statistics. On the benchmark functions F6 and F8, both the CG-GWO algorithm and the WOA algorithm had found the optimal value, but our approach was more concentrated and more stable.

As shown in Table 6, the CG-GWO algorithm showed great superiority on unimodal functions compared with a series of improved algorithms of GWO. Especially on the benchmark functions F1, F2, and F3, the optimal value, average value, worst value and standard deviation are far superior to other improved GWO algorithms in the experiment. The stability of our approach can also be clearly seen on the benchmark functions F4 and F5. On multimodal functions, our approach could find the theoretical optimal value well on the benchmark functions F6, F7, F8 and F10. At the same time, all statistics were better than other optimization algorithms in the experiment. On the benchmark function F9, in addition to the traditional GWO algorithm, other improved algorithms had found the optimal value of the function. But our approach showed greater superiority in comparison. On fixed-dimension multimodal functions, the optimization accuracy of our approach was comparable to that of other improved algorithms in the experiment. Experiments showed that all optimization algorithms had found the optimal value on the benchmark function F12. The GLF-GWO algorithm also showed the same superiority in terms of average and worst value at the same time. But in contrast, our approach was more excellent in standard deviation, and the optimization effect of which was more stable. On the benchmark function F13, the GLF-GWO algorithm and the CG-GWO algorithm had also found the optimal value, but our approach showed more significant concentration and stability.

#### Convergence speed analysis

**Figure 5:**
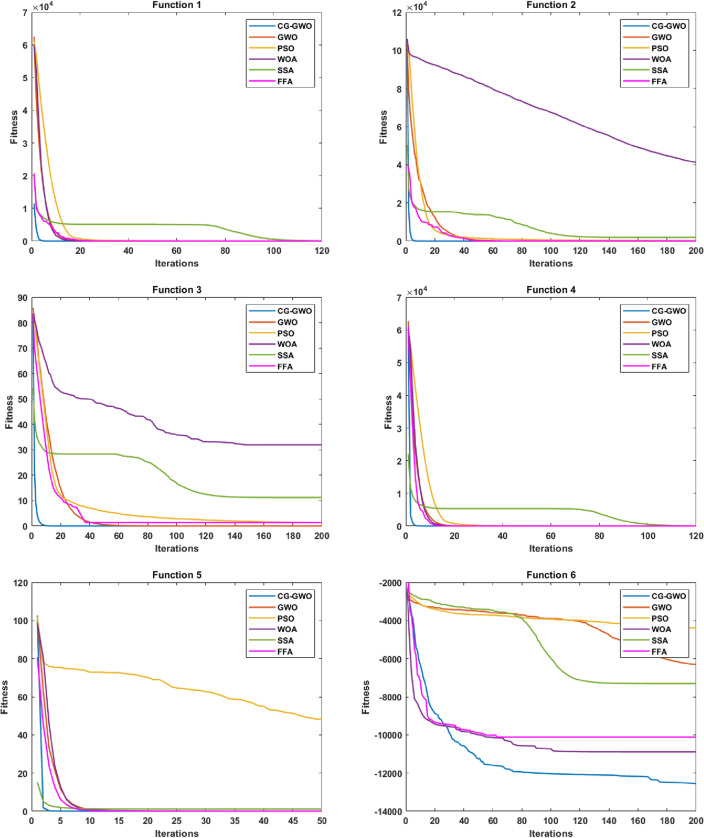

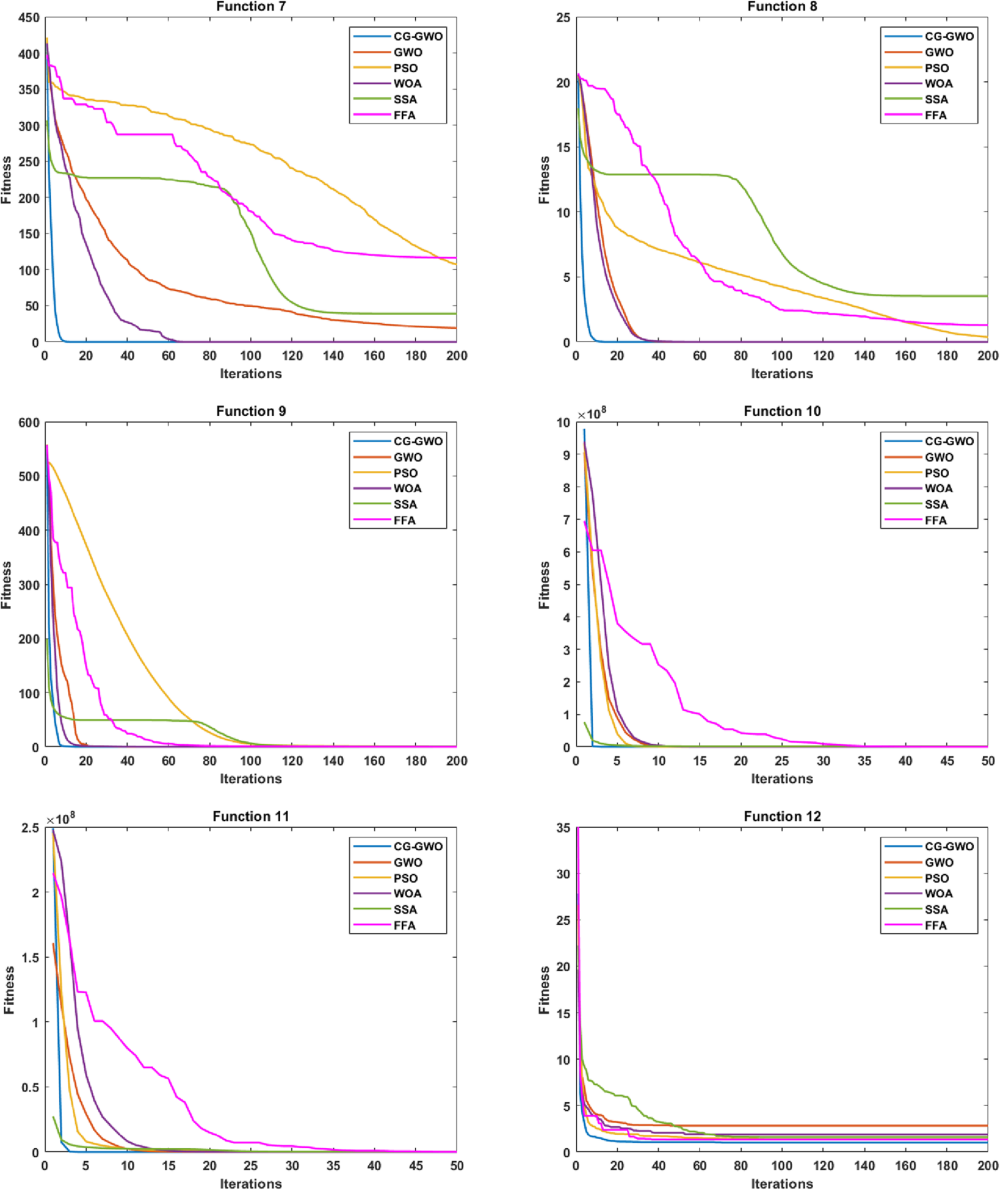

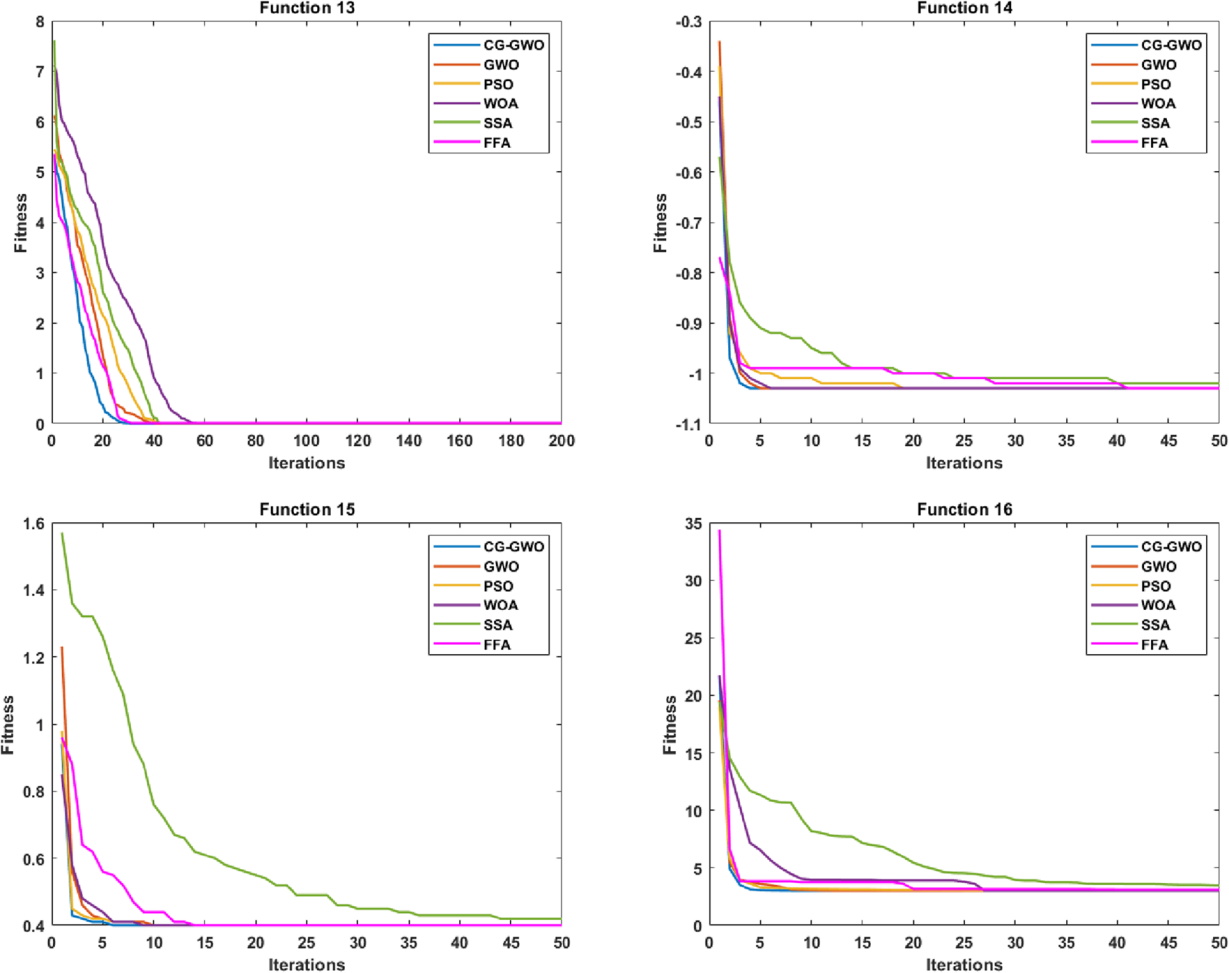
Convergence curve comparison of classic optimization algorithms.

**Figure 6:**
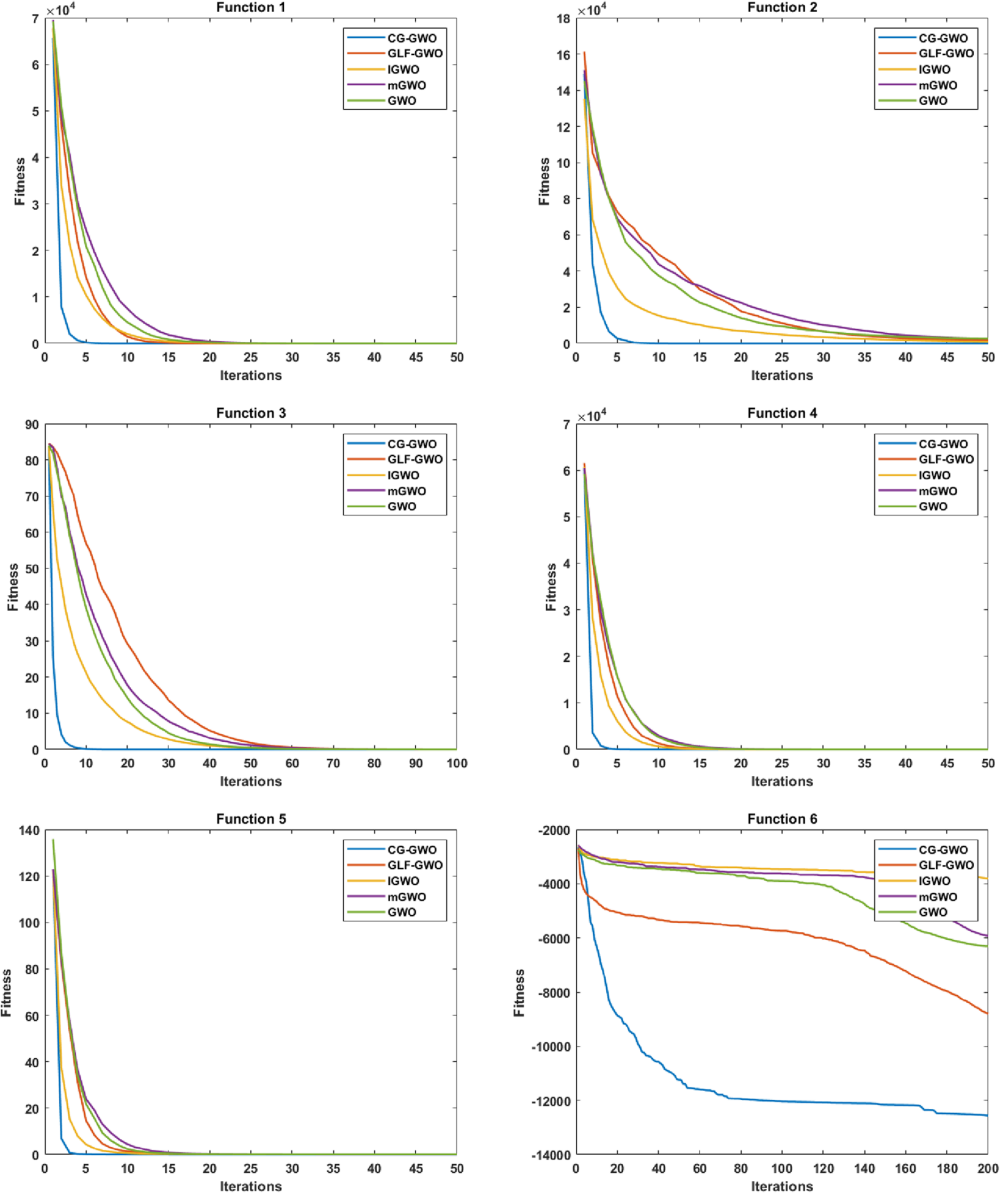

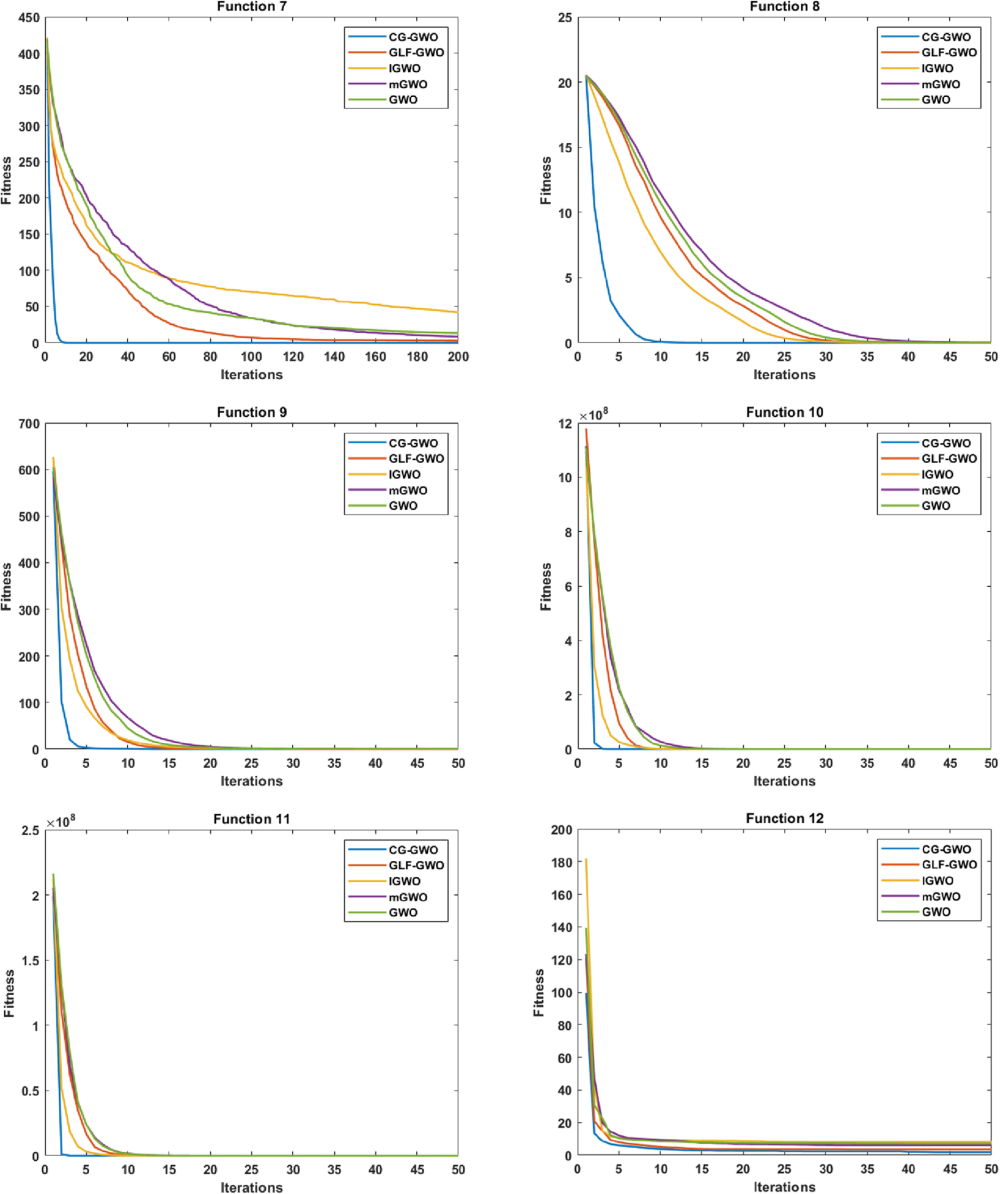

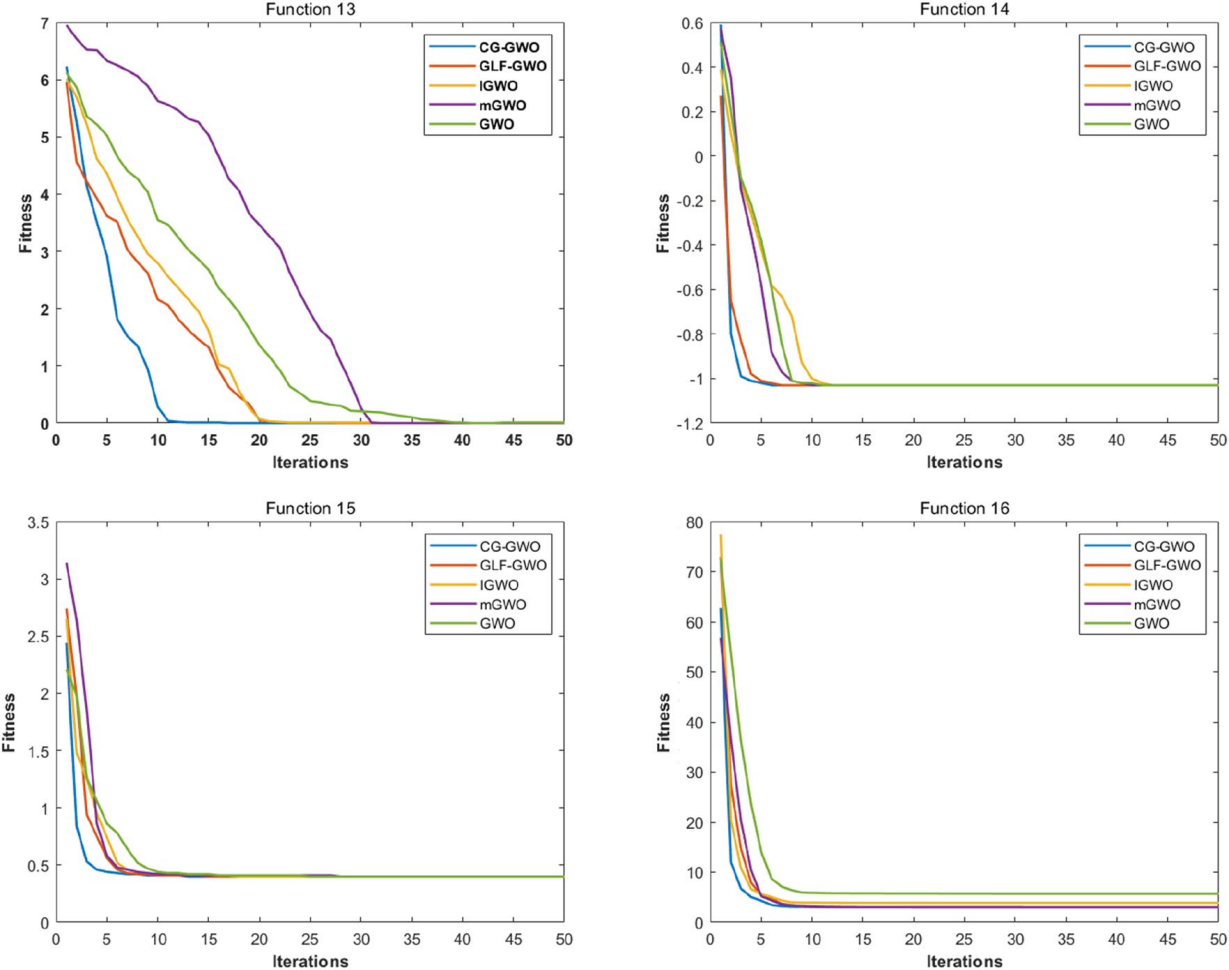
Convergence curve comparison of improved GWO algorithms.

Convergence performance experiments are conducted to observe the convergence effect and convergence speed of the CG-GWO algorithm more intuitively, classical optimization algorithms, and a series of improved algorithms of GWO. The fitness convergence curves of each algorithm on 16 benchmark functions are drawn respectively as shown in Figs. 5 and 6, using the number of iterations as the abscissa and the fitness value as the ordinate.

It can be seen from Fig. 5 that the convergence curve of the CG-GWO algorithm is below other classical optimization algorithms in the experiment. The convergence accuracy and convergence speed on 16 benchmark functions have been significantly improved. For the unimodal functions, as shown in F1 and F4, CG-GWO converged quickly to the optimal value, but SSA still did not reach the optimal value after 100 iterations. As shown in F2 and F3, our approach had been optimized to the optimal value in the 10th iteration. The GWO algorithm converged to the optimal value until the 50th iteration. In contrast, the PSO, WOA, SSA and FFA algorithms still did not reach the optimal value after 200 iterations, and the optimization results of WOA and SSA algorithms were quite different from the optimal value. As shown in F5, PSO did not converge to the optimal value. For the multimodal functions, as shown in F6, GWO and PSO did not tend to converge after 200 iterations. The SSA algorithm converged in the 110th iterations, but which fallen into a local optimum. Both WOA and CG-GWO had a large convergence curve slope at the beginning of the iteration. Although our approach had a slower convergence than WOA in the early stage, the convergence speed and accuracy of which were higher than WOA after 40 iterations. The above comparison also fully reflected the global search ability of our approach. As shown in F7–F8, PSO, SSA and FFA did not converge to the optimal value after 200 iterations. As shown in F9–F11, CG-GWO converged to the optimal value faster than other algorithms. For the fixed-dimension multimodal functions, as shown in F12-F16, all optimization algorithms could converge to close to the optimal value. But in contrast, the CG-GWO algorithm converged faster, and the WOA algorithm converges the slowest. The convergence curve of the WOA algorithm tends to stabilize until the 50th iteration.

It can be seen from Fig. 6 that the CG-GWO algorithm had shown its superiority compared to the traditional GWO algorithm and a series of improved algorithms. On the unimodal functions F1–F5, all algorithms could converge to the optimal value. But our approach could converge to a stationary value faster. On the multimodal functions F6 and F7, our approach could explore closer to the theoretical optimal value after 200 iterations, and the convergence speed of which was also faster. However, other improved algorithms in the experiment either did not converge to a stable value or fall into a local optimal value. On the multimodal functions F8, F9, F10 and F11, all optimization algorithms in the experiment could also converge to the optimal value, but our approach converged more quickly. On the fixed-dimension multimodal function F12 and F16, the convergence speed of all algorithms in the experiment was comparable. However, the convergence accuracy of our approach was higher. Similarly, on the fixed-dimension multimodal function F13–F15, our approach could converge to the optimal value faster than other optimization algorithms in the experiment.

Therefore, our approach is superior to other optimization algorithms in the experiment in terms of convergence accuracy and convergence speed, and the global search ability of that is also significantly improved, which can effectively avoid falling into the local optimal value. Experiments have proved the effectiveness of improved ideas and reflected the superiority of the CG-GWO algorithm in solving more complex optimization problems.

#### Algorithm performance analysis

To evaluate the optimization ability and stability of the improved algorithm, this work draws the box plot^33^ of all algorithms on 16 benchmark functions. Comparative analysis is performed based on the upper four scores, the median, the lower four scores and outliers in the box plot. This work conducts 30 independent experiments on all algorithms. Since experimental results of different algorithms are quite different, different coordinate systems are used for comparison when drawing the box plot to observe the comparison results more intuitively. Experimental results are presented in Figss. 7 and 8.

**Figure 7:**
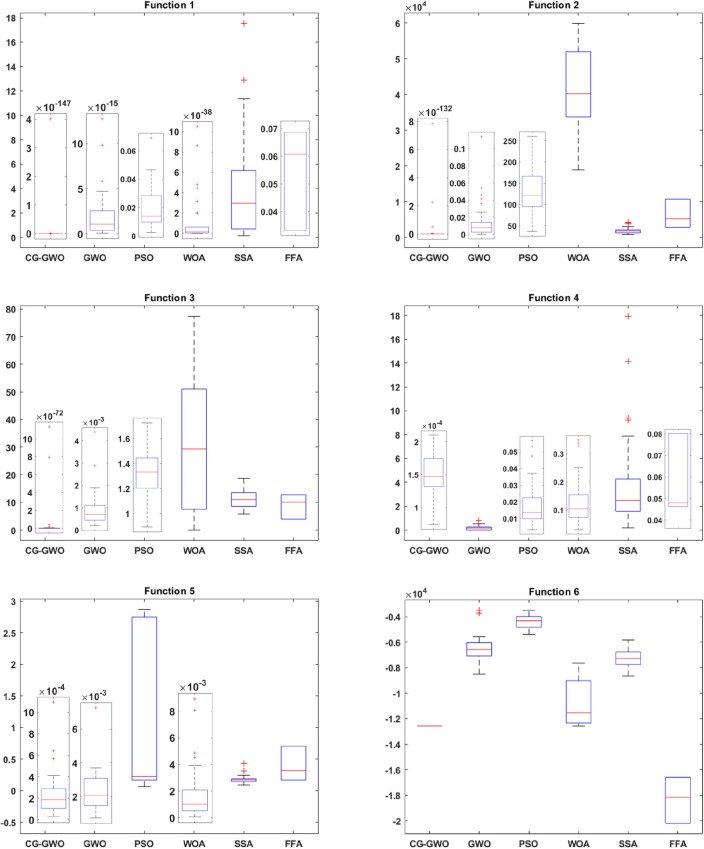

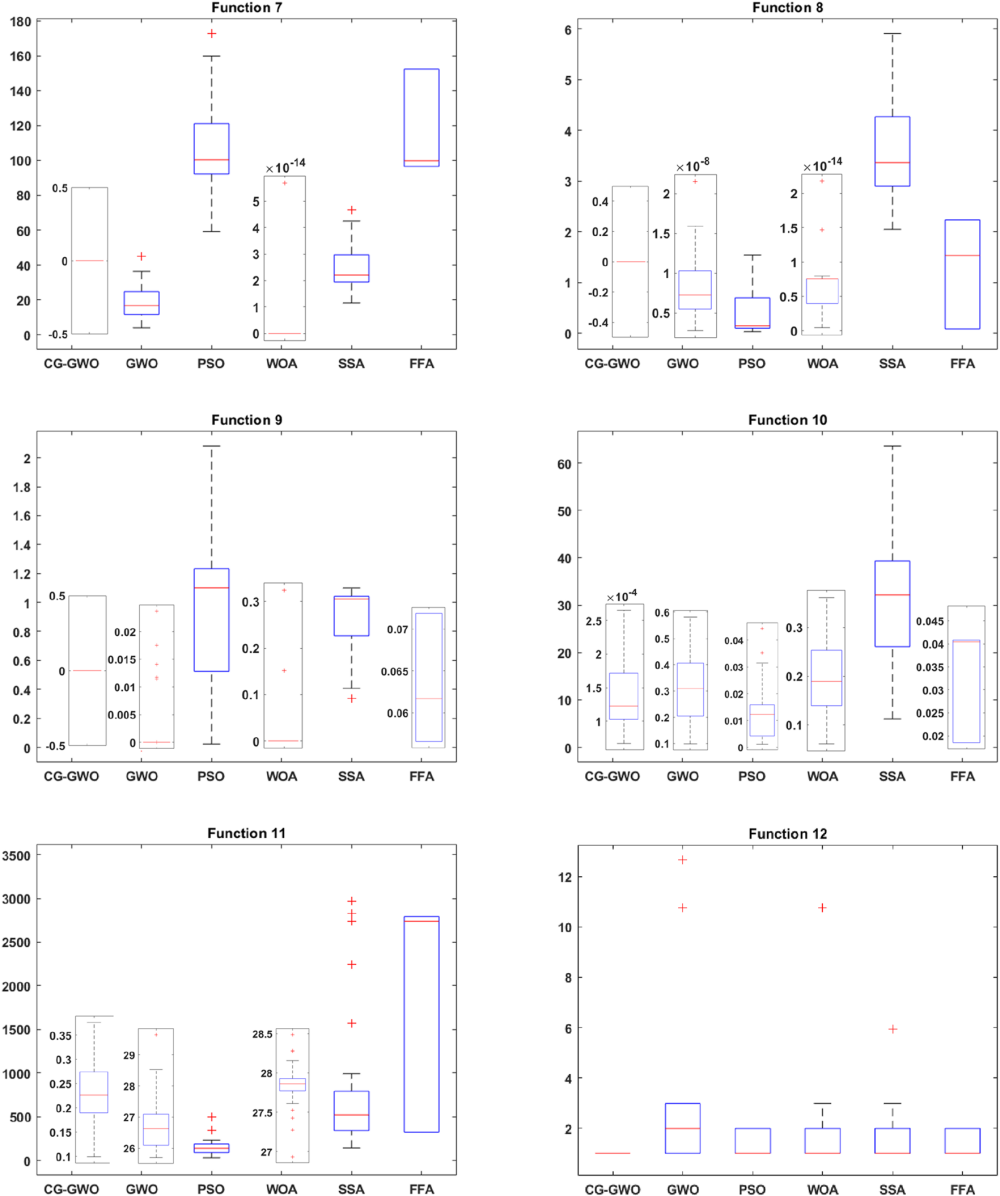

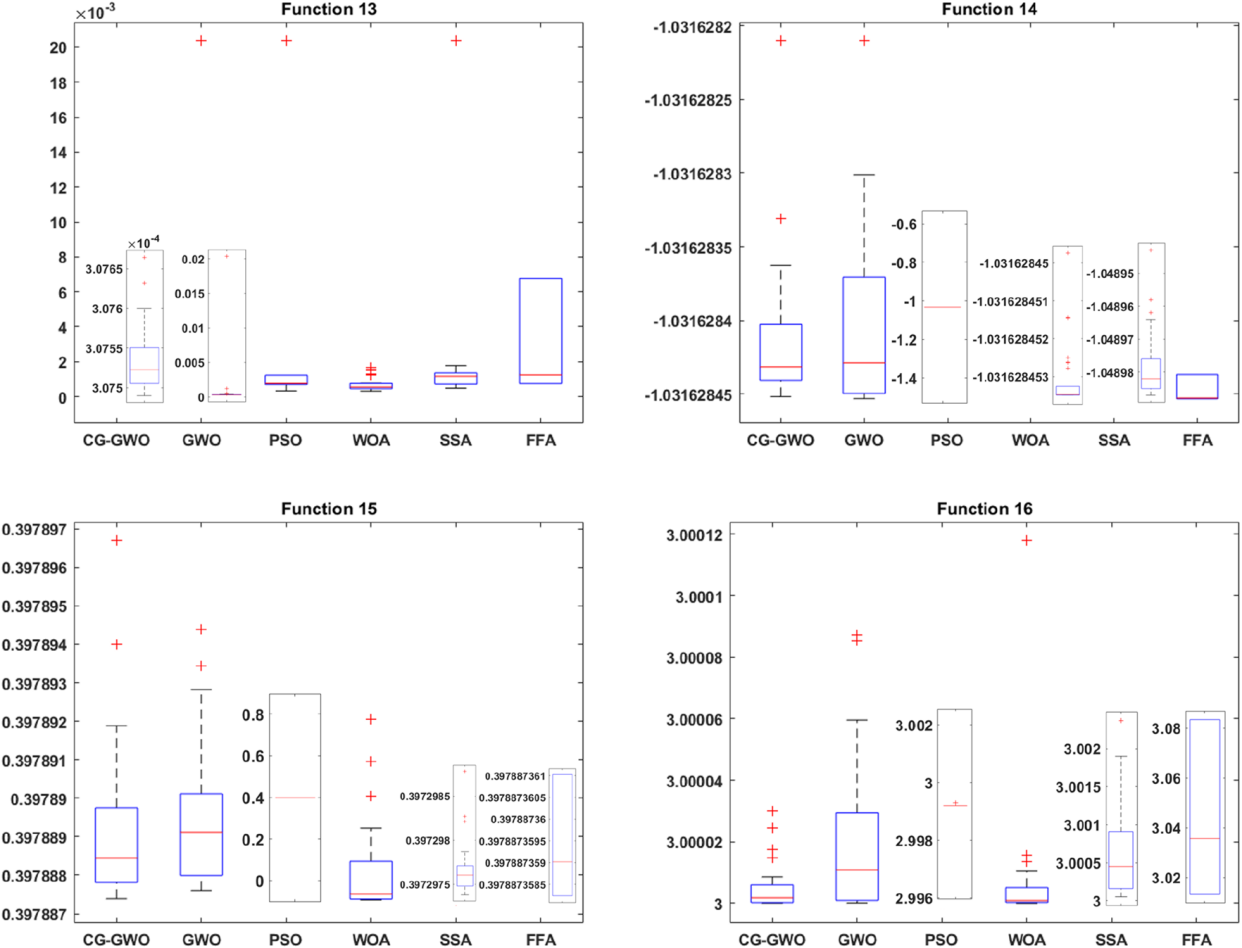
Boxplot comparison of classic optimization algorithms.

**Figure 8:**
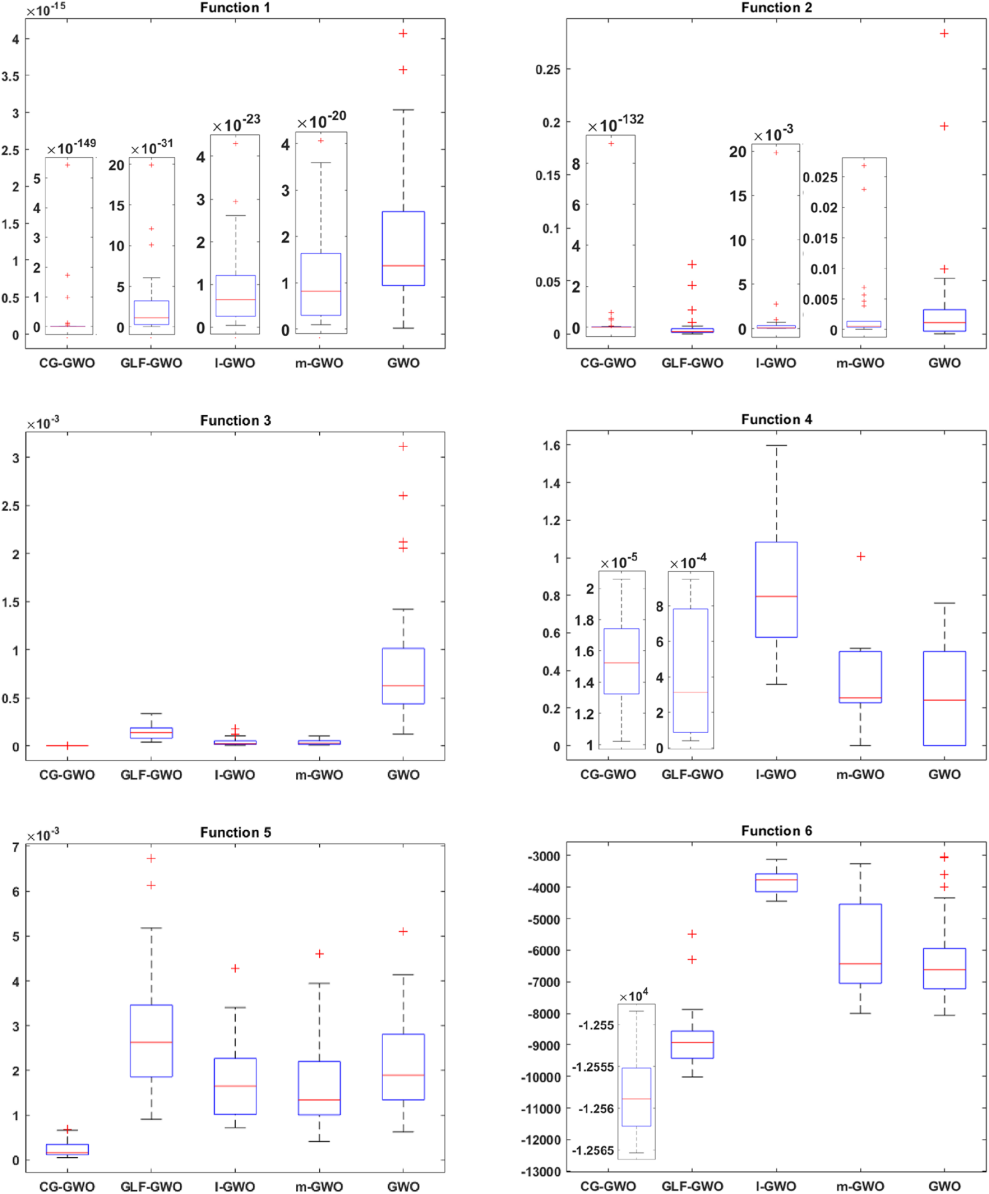

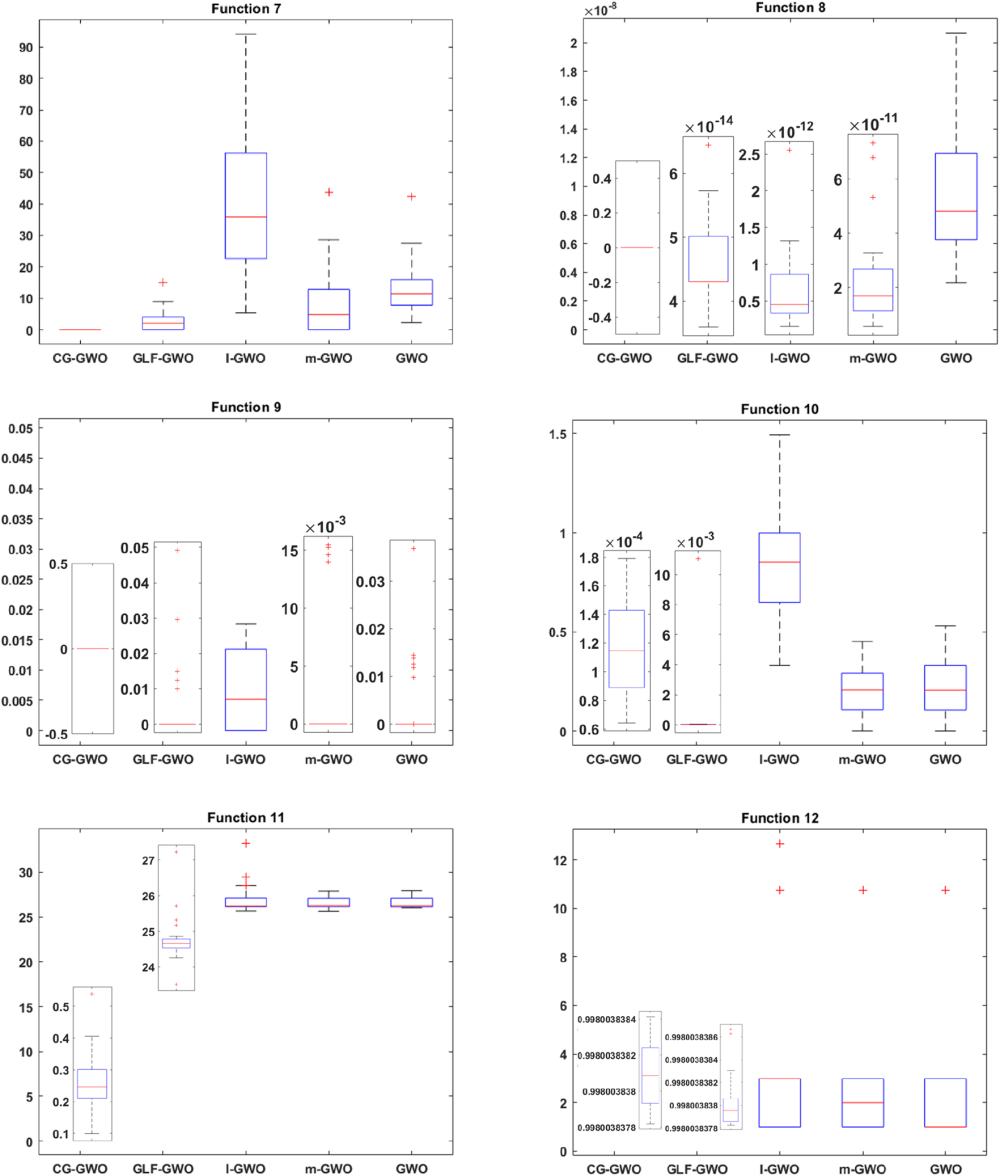

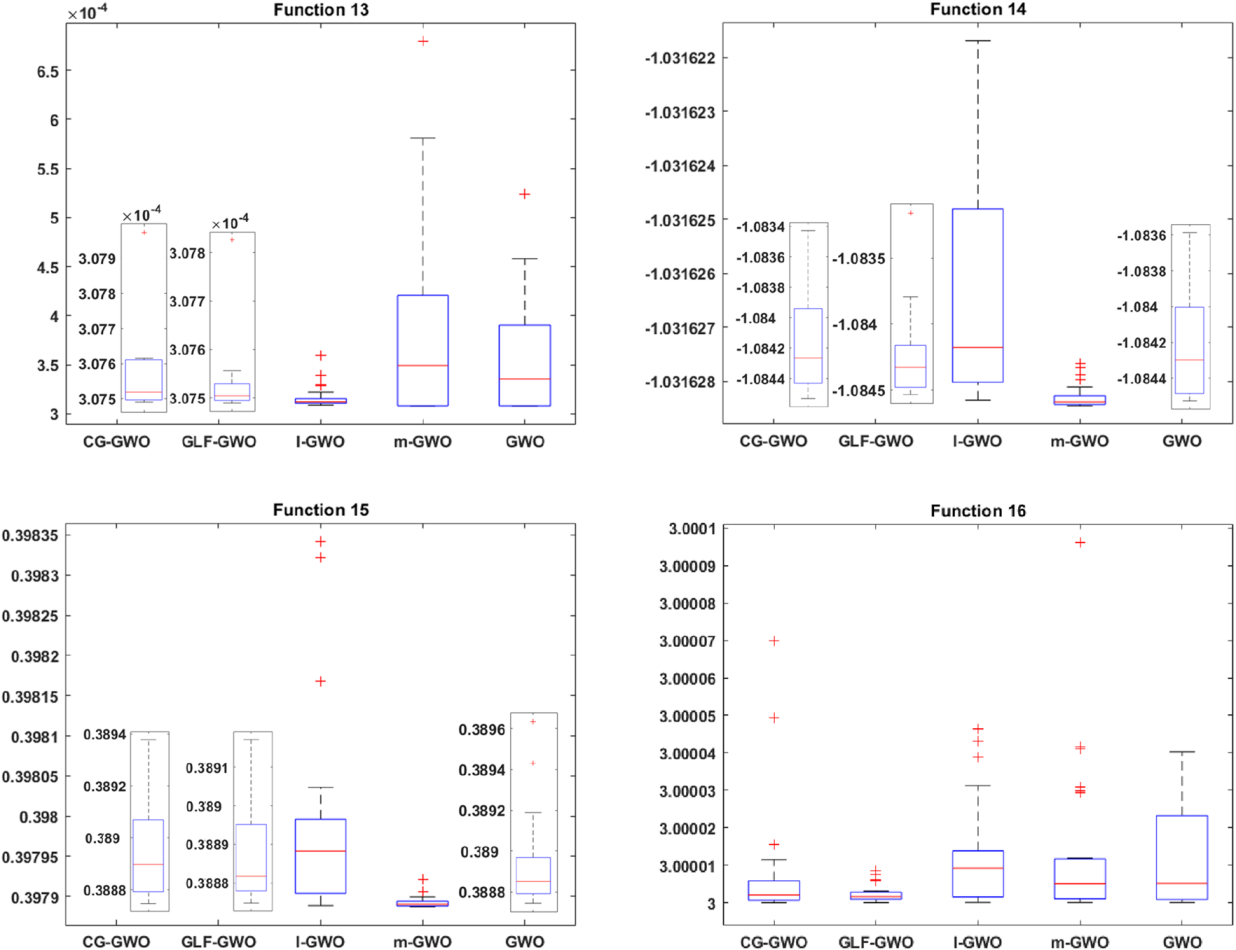
Boxplot comparison of improved GWO algorithms.

It can be seen from Fig. 7 that the CG-GWO algorithm has a weaker dispersion in the optimization process, and the optimization value of which is more concentrated. On the unimodal functions F1–F5, the order of magnitude of the optimization results of our approach was much smaller than other classic optimization algorithms in the experiment. Experimental results reflected the superiority in the optimization accuracy and higher stability of the improved algorithm. On the multimodal functions F6–F11, our approach was more concentrated and has fewer outliers than other classic optimization algorithms in the experiment. Experimental results verified the robustness of our approach in terms of global search ability. On the fixed-dimension multimodal functions F12–F13, the CG-GWO algorithm and the GWO algorithm had similar optimization effects. They were far better than other classic optimization algorithms in the experiment. However, our approach had fewer outliers, more concentrated optimization values and better optimization results. As shown in F14–F16, CG-GWO had fewer outliers and was more stable.

As shown in Fig. 8, most improved GWO algorithms showed superiority compared to the traditional GWO algorithm. On the unimodal functions F1–F5, the CG-GWO algorithm had a better optimization effect. On the benchmark functions F3 and F5, the contrast degree of each algorithm was relatively close, but our approach showed higher convergence stability and had fewer outliers. On the multimodal functions F6–F11, only the superiority of the GLF-GWO algorithm was close to that of our approach. Although the global search ability of the GLF-GWO algorithm was enhanced due to the Levy-flight search mechanism, it produced more outliers. On the fixed-dimension multimodal functions F12–F16, the GLF-GWO algorithm was relatively close to our approach. The GLF-GWO algorithm was better than the CG-GWO algorithm on the range of the optimal value change, but our approach had far fewer outliers. Experimental results embodied the better global search ability and higher stability of our approach.

#### Algorithm runtime analysis

**Figure 9:**
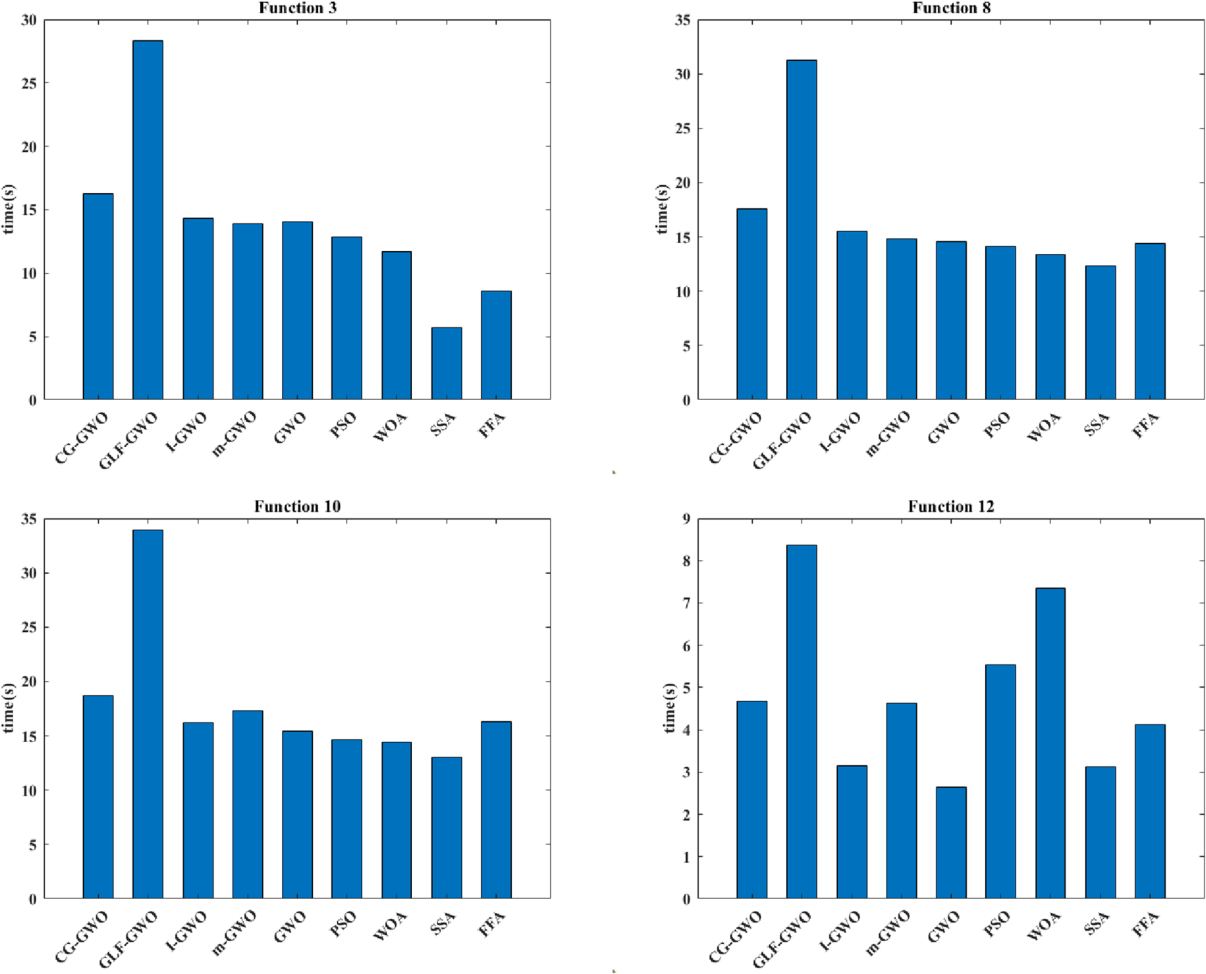
Runtime comparison of algorithms.

To verify the computational effectiveness of the CG-GWO algorithm, this paper compares the runtime of the CG-GWO algorithm with other algorithms. Experimental results are presented in Table 7. To save space, this paper selects four representative functions to draw bar graph. Experimental results are presented in Fig. 9.

**Table 7:** Experimental results comparison of algorithm runtime.

Function	CG-GWO	GLF-GWO	I-GWO	m-GWO	GWO	PSO	WOA	SSA	FFA
F1	16.42	27.63	14.14	13.86	13.99	12.84	12.05	11.73	12.63
F2	20.85	80.11	17.47	16.91	17.09	16.02	15.64	17.02	16.67
F3	16.28	28.32	14.32	13.88	14.03	12.88	11.71	5.71	8.43
F4	16.34	31.3	14.19	13.97	14.01	12.87	12.12	11.38	12.42
F5	17.13	33.74	14.38	14.07	14.15	13.53	12.27	10.42	12.65
F6	16.92	34.91	14.25	14.01	14.17	13.42	12.29	10.39	11.73
F7	16.16	35.15	14.09	15.05	13.72	12.79	11.69	11.9	11.42
F8	17.58	31.25	14.54	14.86	14.55	14.18	13.38	12.38	14.25
F9	18.36	42.09	14.67	14.65	14.78	14.07	12.88	12.49	13.75
F10	18.7	33.94	15.18	16.29	15.4	14.69	14.42	13.02	15.36
F11	16.64	40.04	14.33	14.07	14.15	13.35	12.21	10.46	11.72
F12	4.68	8.36	3.15	4.63	2.64	5.53	7.35	3.13	4.13
F13	5.68	8.36	4.15	12.14	12.11	12.19	7.35	3.13	7.62
F14	2.27	7.91	2.41	2.63	2.64	5.53	1.06	2.49	2.56
F15	2.3	2.02	2.42	1.11	1.11	1.45	1.59	1.09	1.52
F16	2.67	2.56	2.54	2.13	2.14	1.66	1.79	1.36	1.84

As shown in Table 7 and Fig. 9, the CG-GWO algorithm has Cauchy-Gaussian mutation in the improved strategy, which will consume more time. In F3, F8 and F10, the runtime of CG-GWO was only lower than GLF-GWO. However, we can see that the increase of time consumption was not too great. We can accept this change in practical application. In F12, the runtime of CG-GWO is medium. So, the CG-GWO algorithm has certain computational validity.

Therefore, the CG-GWO algorithm has a more stable convergence ability compared with classic optimization algorithms and a series of improved GWO algorithms. Our approach shows its superiority in the optimization accuracy and the degree of dispersion. Experimental results prove that our approach has better optimization ability and stable performance.

#### Case study of real-world application

IN this section, the performance of the nine mentioned algorithms, PSO, WOA, SSA, FFA, GWO, I-GWO, m-GWO, GLF-GWO and CG-GWO, is evaluated in engineering real application: pressure vessel design^34^.

**Figure 10:**
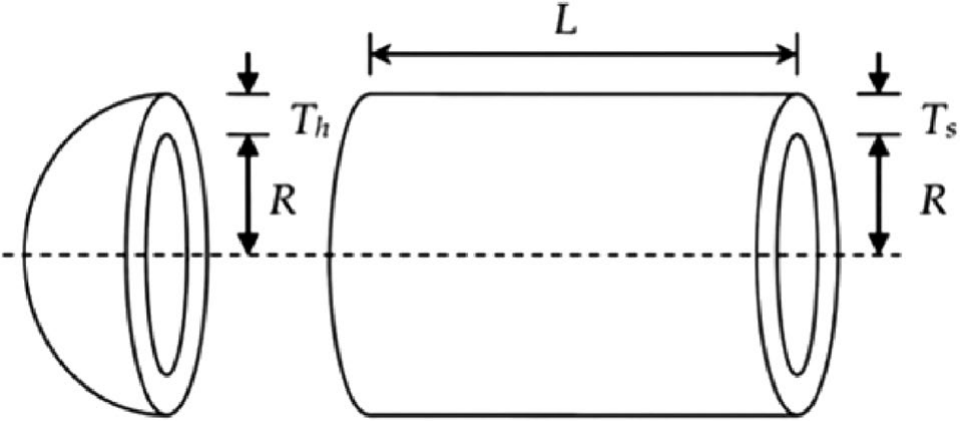
Pressure vessel design problem.

Pressure vessels are usually spherical or cylindrical in shape. Cylindrical vessels may be oriented vertically or horizontally. Vertical vessels have many uses: Fractionating Towers, Contactor Towers, Reactors and Vertical Separators. This problem works to reduce the overall cost of material, formation, and welding of the cylindrical pressure vessel reinforced at both ends by hemispherical heads as shown in Fig. 10. The mathematical formulation of pressure vessel design is described as follows:

15 $$\begin{gathered} \min f(T_{s} ,T_{h} ,R,L) = 0.6224T_{s} RL +1.7781T_{h} R^{2} +3.1661T_{s}^{2} L +19.84T_{h}^{2} L \hfill \\ s.t.\left\{ \begin{gathered} g_{1} = - T_{s} + 0.0193R \le 0 \hfill \\ g_{2} = - T_{h} + 0.0095R \le 0 \hfill \\ g_{3} = - \pi R^{2} L - 4/3\pi R^{3} + 1296000 \le 0 \hfill \\ g_{4} = L - 240 \le 0 \hfill \\ \end{gathered} \right. \hfill \\ \end{gathered}$$

where $$0 \le T_{s} ,T_{h} \le 99$$ and $$10 \le R,L \le 200.$$

The pressure vessel design problem is one of the most common problems. Researchers have used optimization problems in many studies to confirm the efficacy of their new optimization algorithms. The comparison of the optimal results obtained for the pressure vessel design problem by CG-GWO and other algorithms mentioned above is presented in Table 8. According to the cost results obtained for the pressure vessel design problem in Table 8, CG-GWO reported the lowest of 5884.6849. And the optimum parameters are 0.779216, 0.396459, 40.265625 and 200.000000.

**Table 8:** Optimization results of the pressure vessel design problem.

Algorithm	Optimal values for variables				Optimum cost
	$T_{s}$	$T_{h}$	R	L	
PSO	0.784269	0.376984	39.462015	200.000000	5896.7468
WOA	0.826300	0.426981	41.035792	178.531864	5966.4823
SSA	0.805623	0.402982	40.539225	186.265325	5915.6825
FFA	0.789212	0.396525	40.365296	200.000000	5887.6842
GWO	0.776351	0.384192	40.359152	200.000000	5891.5843
I-GWO	0.743695	0.394623	39.595432	196.252352	5896.5222
m-GWO	0.763152	0.403418	41.684583	200.000000	5888.5622
GLF-GWO	0.786928	0.406852	40.165236	200.000000	5886.1356
CG-GWO	0.779216	0.396459	40.265625	200.000000	**5884.6849**

Significant values are in bold.

The comparison of the statistical results for the pressure vessel design problem over 50 independent runs is shown in Table 9. It can be seen from results that the stability of CG-GWO is better, which presented excellent results in terms of Ave and Std values.

**Table 9:** Statistical results of CG-GWO and other optimization algorithms for the pressure vessel design problem.

Algorithm	Best	Ave	Worst	Std
PSO	5896.7468	5897.5922	5923.3156	13.6238
WOA	5966.4823	5968.1652	6000.2668	15.6965
SSA	5915.6825	5920.2392	5986.2552	8.6437
FFA	5887.6842	5888.6462	5892.5463	9.6562
GWO	5891.5843	5895.2652	5900.2623	10.6981
I-GWO	5896.5222	5898.2653	5900.1852	9.6523
m-GWO	5888.5622	5890.4652	5896.2553	8.6521
GLF-GWO	5886.1356	5887.1625	5889.2656	5.6984
CG-GWO	5884.6849	5885.2682	5889.6563	**3.4662**

Significant values are in bold.

## Conclusion and future works

This work proposed CG-GWO algorithm aiming at the slow convergence and easy to fall into local optimal problems of traditional Grey Wolf Optimization algorithm. The Cauchy-Gaussian mutation operator was introduced, which acted on the leader wolves. The dominant degree of Cauchy mutation and Gaussian mutation in the algorithm was dynamically adjusted to improve the global search ability of the algorithm according to the current iteration period. At the same time, the greedy mechanism was added to perform Cauchy-Gaussian mutation in the lead wolves. Outstanding individuals during mutation were retained to avoid the high diversity of the algorithm and ensured the convergence speed of the algorithm. CG-GWO added an improved search strategy to avoid the algorithm from falling into the local optimum and improve the convergence accuracy of the algorithm. In the improved strategy, new solutions were generated around random solutions or optimal solutions. Experimental results showed that our approach effectively improved the accuracy and speed of convergence. In the accuracy experiments, CG-GWO showed several times superiority. The convergence performance was also able to converge to the optimal value relatively quickly, while the stability of the algorithm was also shown to be high by the box plot. Although CG-GWO did not have much advantage in terms of runtime, the superiority of the algorithm was also demonstrated by the comparison of the pressure vessel design problem.CG-GWO was able to find the optimal value more consistently than other algorithms mentioned in the paper. In conclusion, CG-GWO showed good optimization ability and stability on unimodal functions, multimodal functions, and fixed-dimension multimodal functions, which could effectively avoid falling into the local optimum and expand the individual search space.

Although CG-GWO algorithm showed good convergence accuracy, convergence speed and stability in most cases, its stability is slightly poor in some specific situations. At the same time, Cauchy-Gaussian mutation will take more time, which is also a limitation of the algorithm because of its long running time. The proposed algorithm has some limitations in practical application. If the scale of the problem is too large, the calculation of $X_{avg} (t)$ in the improved search strategy is relatively more complicated, which will affect the optimization progress of the algorithm. Also, if multiple variables in the actual problem affect each other, it is difficult to make the selection of the leader wolves, and the determination of variable $\sigma$ in the Cauchy-Gaussian mutation will become complicated, which also bring great challenges to the proposed algorithm. In the future work, we should pay attention to the stability improvement of the algorithm and improve the efficiency of the algorithm. The CG-GWO algorithm will be applied to more complex practical engineering optimization problems to help optimizers determine the final optimization plan more quickly and accurately.

## Data Availability

The datasets generated and/or analyzed during the current study are not publicly available due that the benchmark functions used in the article have been explained in the experimental part but are available from the corresponding author on reasonable request.

## References

[1] Mirjalili S, Mirjalili M, Lewis A (2014). Grey Wolf optimizer. Adv. Eng. Softw..

[2] Kennedy, N., Eberhart, C. Particle swarm optimization. In *Proceedings of ICNN'95—International Conference on Neural Networks*, vol. 1–6 1942–1948 (2002).

[3] Mirjalili S, Lewis A (2016). The Whale Optimization algorithm. Adv. Eng. Softw..

[4] Xue J, Shen B (2020). A novel swarm intelligence optimization approach: Sparrow search algorithm. Syst. Sci. Control Eng..

[5] Salgotra R, Singh U, Sharma S (2020). On the improvement in Grey Wolf Optimization. Neural Comput. Appl..

[6] Miao D, Chen W, Zhao W (2020). Parameter estimation of PEM fuel cells employing the hybrid Grey Wolf Optimization method. Energy.

[7] Kulkarni O, Kulkarni S (2018). Process parameter optimization in WEDM by Grey Wolf optimizer. Mater. Today Proc..

[8] Luo K, Zhao Q (2019). A binary grey wolf optimizer for the multidimensional knapsack problem. Appl. Soft Comput..

[9] Zewen L, Yichao H, Ya L (2020). A hybrid grey wolf optimizer for solving the product knapsack problem. Int. J. Mach. Learn. Cybern..

[10] Kamboj K, Bath K, Dhillon S (2015). Solution of non-convex economic load dispatch problem using Grey Wolf Optimizer. Neural Comput. Appl..

[11] Kadali S, Rajaji L, Moorthy V (2017). Economic generation schedule on thermal power system considering emission using grey wolves optimization. Energy Procedia.

[12] Qiu J, Chen M, Wei Z (2020). Planning and optimal scheduling method of regional integrated energy system based on Gray Wolf Optimizer algorithm. IOP Conf. Ser. Earth Environ. Sci..

[13] Yang Z, Liu C (2018). A hybrid multi-objective gray wolf optimization algorithm for a fuzzy blocking flow shop scheduling problem. Adv. Mech. Eng..

[14] Jiang T (2018). A hybrid Grey Wolf optimization for job shop scheduling problem. Int. J. Comput. Intell. Appl..

[15] Zhang X, Liu Z, Miao Q (2018). An optimized time varying filtering based empirical mode decomposition method with grey wolf optimizer for machinery fault diagnosis. J. Sound Vib..

[16] Zeng B, Guo J, Zhu W (2019). A transformer fault diagnosis model based on hybrid Grey Wolf Optimizer and LS-SVM. Energies.

[17] Jiang Y (2020). Fault diagnosis of subway plug door based on Isomap and GWO-SVM. ICIEA.

[18] Emary E, Zawbaa M, Hassanien E (2016). Binary Grey Wolf optimization approaches for feature selection. Neurocomputing.

[19] Pei H, Pan J, Chu S (2020). Improved binary Grey Wolf optimizer and its application for feature selection. Knowl.-Based Syst..

[20] Kitonyi M, Segera R (2021). Hybrid gradient descent Grey Wolf optimizer for optimal feature selection. Biomed. Res. Int..

[21] Kumaran N, Vadivel A, Kumar S (2018). Recognition of human actions using CNN-GWO: A novel modeling of CNN for enhancement of classification performance. Multimedia Tools Appl..

[22] Yao X, Zhiyuan L, Liu L (2019). Multi-threshold image segmentation based on improved Grey Wolf optimization algorithm. IOP Conf. Ser. Earth Environ. Sci..

[23] Bharanidharan N, Harikumar R (2020). Modified Grey Wolf randomized optimization in dementia classification using MRI images. IETE J. Res..

[24] Long W, Jiao J, Liang X (2018). Inspired grey wolf optimizer for solving large-scale function optimization problems. Appl. Math. Model..

[25] Mittal N, Sohi S, Singh U (2016). Modified Grey Wolf optimizer for global engineering optimization. Appl. Comput. Intell. Soft Comput..

[26] Gupta S, Deep K (2019). Enhanced leadership-inspired grey wolf optimizer for global optimization problems. Eng. Comput..

[27] Bansal C, Singh S (2020). A better exploration strategy in Grey Wolf Optimizer. J. Ambient. Intell. Humaniz. Comput..

[28] Mirjalili S, Saremi S, Mirjalili M (2016). Multi-objective grey wolf optimizer: A novel algorithm for multi-criterion optimization. Expert Syst. Appl..

[29] Gharehchopogh S (2022). An improved tunicate swarm algorithm with best-random mutation strategy for global optimization problems. J. Bionic Eng..

[30] Bengag A, Bengag A, Elboukhari M (2020). A Novel Greedy forwarding mechanism based on density, speed and direction parameters for Vanets. Int. J. Interact. Mobile Technol. (iJIM).

[31] Heidari A, Pahlavani P (2017). An efficient modified grey wolf optimizer with Lévy flight for optimization tasks. Appl. Soft Comput..

[32] Shayanfar H, Gharehchopogh S (2018). Farmland fertility: A new metaheuristic algorithm for solving continuous optimization problems. Appl. Soft Comput..

[33] Xiaobing Y, Chenliang L, Chen H (2019). Evaluate the effectiveness of multiobjective evolutionary algorithms by box plots and fuzzy TOPSIS. Int. J. Comput. Intell. Syst..

[34] Yang S, Xingshi H (2013). Firefly algorithm: Recent advances and applications. Int. J. Swarm Intell..

